# The continuing significance of chiral agrochemicals

**DOI:** 10.1002/ps.8655

**Published:** 2025-01-17

**Authors:** Peter Jeschke

**Affiliations:** ^1^ Heinrich‐Heine‐University Düsseldorf Institute of Organic Chemistry and Macromolecular Chemistry Duesseldorf Germany

**Keywords:** chiral agrochemicals, structure–activity relationship, herbicides, fungicides, insecticides, acaricides, nematicides, mode of action, physicochemistry, selectivity, resistance

## Abstract

Chemical crop protection is one of the most cost‐effective methods for agriculture, as crop failures can be prevented, and sustainable growth can be enabled regardless of the seasons. Agricultural production must be significantly increased in the future to meet the food needs of a growing world population. However, the continued loss of established active ingredients due to consumer perceptions, changing needs of farmers and ever‐changing regulatory requirements is higher than annually new active ingredients introduced to the market. The development of innovative active ingredients is therefore essential to continuously improve the selectivity, efficacy and favorable environmental profile of agrochemicals. Molecules with stereogenic centers can be considered here, as they often have different properties than non‐chiral molecules. Natural products and their congeners are still a valuable source of inspiration for chiral agrochemicals. However, only a few novel chiral agrochemicals are currently produced on an industrial scale as pure stereoisomers or in enriched form. As of 2018, around 43% of the 35 chiral products introduced to the market (herbicides, fungicides, insecticides, acaricides, and nematicides) contain one or more stereogenic centers in the molecule, and almost 69% of them have been marketed as racemic mixtures of enantiomers or stereoisomers. Surprisingly, the proportion of chiral agrochemicals is in the same order of magnitude as in the time frame from 2007 to 2017 with around 42%, respectively. This report therefore provides an overview of the continued importance of chiral agrochemicals brought to market in the last 6 years and describes the inherent related challenges of modern agrochemicals through the management of key aspects arising from innovative crop protection products. © 2025 The Author(s). *Pest Management Science* published by John Wiley & Sons Ltd on behalf of Society of Chemical Industry.

## Graphical abstract

In the time frame 2018–2023, around 43% of the 35 chiral agrochemicals introduced to the market (herbicides, fungicides, insecticides, acaricides, and nematicides) contain one or more stereogenic centers in the molecule, and almost 69% of them have been marketed as racemic mixtures of enantiomers or stereoisomers.

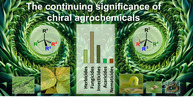

## 1 INTRODUCTION

Today, the crop protection industry faces enormous challenges to guarantee sustainability and technical progress. The focus is on novel agrochemicals with optimal efficacy, lower application rate in the field, increased selectivity, favorable toxicological and ecological safety, improved user‐friendliness and better cost‐effectiveness. The continuous loss of older active ingredients (a.i.s) in crop protection due to consumer perception, changing needs of growers and ever‐changing regulatory requirements is far higher than the number of them being introduced on the market. Therefore, there is an urgent need to develop novel innovative products that can offer these improved efficacy, selectivity and favorable environmental profiles. One strategy to achieve the ambitious goals is to design new a.i.s with increasing molecular complexity, caused by the presence of one or more stereogenic centers in the molecule. For example, natural products and their congeners are still an important source of inspiration for the development and search for new a.i.s.^1^ It has been shown that in many racemic compounds only one enantiomer is biologically active, or that one enantiomer is significantly more active than the other enantiomer.^2^ Despite the enormous advances made in catalytic asymmetric processes in recent years, only a few agrochemicals are produced on an industrial scale as pure stereoisomers or enriched stereoisomers.

## 2 STEREOCHEMISTRY APPROACH IN MODERN AGROCHEMISTRY

The stereochemistry approach has been mainly used for drug design in medicinal chemistry and has been described considering both marketed products and research examples in various articles^3^, ^4^, ^5^ and book chapters.^6^ However, mostly older comprehensive articles^7^, ^8^, ^9^, ^10^ and reviews have been published on chiral agrochemicals.^11^, ^12^, ^13^, ^14^, ^15^, ^16^

### 2.1 Importance of chirality in modern agrochemicals

Since the 1990s, the enantioselective effects and related physicochemical properties of chiral agrochemicals have received increasing attention.^17^ Chiral agrochemicals with high optical purity can have advantages such as lower toxicity, higher efficiency and reduced application rates and have thus become important for the search for new a.i.s. Modern agrochemicals currently on the market are more sophisticated in their molecular structures featuring one or several stereogenic centers in the molecule. Stereoisomers are known to have distinct absorption, distribution, metabolism, and excretion (ADME) characteristics.^18^ Currently, the evaluation of pesticide‐likeness is mainly based on ADME and in addition toxicity, the so‐called ADME‐T property concepts of agrochemicals.^19^ In recent years, the concept of target‐based screening programs has remarkably influenced the design of molecules fitting optimal to chiral cellular receptors or binding proteins.^20^ Furthermore, the increasing knowledge available about biochemical pathways, the chemical structure of chiral metabolites, and ligand receptor interactions can support the efficient design for optimal a.i.s in crop protection.

### 2.2 Technical manufacturing of chiral agrochemicals

While in the past selective manufacturing of stereoisomers or their separation on an industrial scale was often difficult, inefficient and expensive, today there are various methods to provide chiral agrochemicals or to analyze them with good resolution and sensitivity.^21^, ^22^ Most technical manufacturing (Methods I–V)^23^ can also be exemplified by the chiral agrochemicals of the last 6 years (2018–2023), which have been produced on an industrial scale with the required efficiency.

#### 2.2.1 Method I. Separation of stereoisomers

Based on publications of the last 3 years, chromatography, electrophoresis and membrane separation are still typical technologies for chiral separation and analysis of chiral compounds. Today, the search for new chiral stationary phases (CSPs) and chiral selectors is a major direction of chiral chromatography, electrophoresis, and membrane separation.^24^ High‐performance liquid chromatography (HPLC), gas chromatography (GC), and supercritical fluid chromatography (SFC) are still the primary methods for the separation and analysis of stereoisomers. Over the last few decades, counter‐current chromatography (CCC) has been successfully applied to the area of chiral separations. It provides an important approach to obtain pure enantiomers, particularly in preparative application because of its unique advantages of high‐load capacity, low solvent consumption, and easy scale‐up.^25^

A method for producing the intermediate (*R*)‐1,1,3‐trimethyl‐4‐aminoindane for the fungicide inpyrfluxam (see Section 5.1.1) by using d‐tartaric acid (methanol, toluene, water, 5 h, 40 °C) includes different steps: (a) optically resolving 1,1,3‐trimethyl‐4‐aminoindane to obtain both the (*R*)‐ and (*S*)‐enantiomers, (b) racemization of the (*S*)‐enantiomer obtained in the step (a) or (c) so as to obtain 1,1,3‐trimethyl‐4‐aminoindane, and (c) optically resolving the 1,1,3‐trimethyl‐4‐aminoindane obtained in step (b) so as to obtain the (*R*)‐ and (*S*)‐enantiomers.^26^

For the enantioseparation of the fungicide fluindapyr (see Section 5.1.2) three kinds of chiral chromatographic columns were used. For example, fluindapyr enantiomers have been separated by Daicel Chiralpak AD‐3R (amylose tris(3,5‐dimethylphenylcarbamate)), Daicel Chiralpak OX‐3R (cellulose tris(4‐chloro‐3‐methylphenylcarbamate)), and Daicel Chiralpak IK‐3 (cellulose tris(3‐chloro‐5‐methylphenylcarbamate)). Finally, due to the favorable separation effect, Daicel Chiralpak IK‐3 has been chosen for further studies (Supporting Information Table S1).^27^

The chiral separation and detection of the fungicide mefentrifluconazole (see Section 5.4.1) enantiomers has been performed on a Waters ACQUITY H‐Class PLUS ultraperformance liquid chromatography (UPLC) system tandem with a Waters Xevo TQ‐S Micro MS/MS (Milford, MA, USA) with a Superchiral IG‐3 column (amylose‐tris(3‐chloro‐5‐methylphenylcabamate)) (Table S1).^28^

The chiral separation and preparation method of the insecticide fluxametamide (see Section 6.3.1) enantiomers was developed based on ultraperformance SFC tandem mass spectrometry (SFC‐MS/MS) (Table S1).^29^

#### 2.2.2 Method II. Use of chiral building blocks

The chiral pool of useful starting compounds contains relatively inexpensive, commercially available chiral natural products (e.g., carbohydrates, terpenes, alkaloids, hydroxyl‐ and amino acids), available with high enantiomeric excess (ee) and in all enantiomeric forms. Among the most important chiral building blocks, proteinogenic and non‐proteinogenic (*S*)‐amino acids play a dominant role and these are available commercially.

In the case of the fungicide florylpicoxamid (see Section 5.2.2), the key intermediate (*S*)‐1,1‐bis(4‐fluorophenyl)propan‐2‐yl l‐alaninate can be prepared starting from (3*S*,6*S*)‐3,6‐dimethyl‐1,4‐dioxane‐2,5‐dione, the dimer of (*S*)‐2‐hydroxypropanoic acid (lactic acid), a raw material available by bacterial fermentation of carbohydrates^30^ and a *tert*‐butyloxycarbonyl (Boc)‐protected amino acid such as (*S*)‐alanine.^31^

#### 2.2.3 Method III. Catalytic asymmetric synthesis

So far, catalytic asymmetric catalysis has proven to be a versatile tool for the enantioselective synthesis of different chiral agrochemicals^32^ and novel drugs in medicinal chemistry.^33^ Therefore, the use of chiral catalysts to transfer and enhance chirality in chemical reactions is also a priority in manufacturing of current active chiral ingredients. Table S2 lists catalytic asymmetric syntheses of the following agrochemical intermediates and final products.

For example, at the beginning of the production process of the herbicide cinmethylin (see Section 4.2.1), α‐terpinene is used, which is converted in the presence of AD‐mix β to (1*S*,2*R*)‐4‐isopropyl‐1‐methylcyclohex‐3‐ene‐1,2‐diol, serving as further stereoisomeric precursor.^34^ AD‐mix is a commercially available mixture of reagents that acts as an asymmetric catalyst for various chemical reactions, including the Sharpless asymmetric dihydroxylation of alkenes.

In the preparation of the chiral herbicide tetflupyrolimet (see Section 4.3.1), the ketone function of the precursor 2‐(benzyl(methyl)amino)‐1‐(3‐(trifluoromethyl) phenyl)ethane‐1‐one‐hydrochloride is stereoselectively reduced to the (*R*)‐enantiomeric alcohol intermediate.^35^, ^36^

During the manufacturing of the fungicide and nematicide cyclobutrifluram (see Section 5.3.1 and 8.1), the intermediate *N*‐(2‐(2,4‐dichlorophenyl)cyclobut‐1‐en‐1‐yl) acetamide is subjected to an enantioselective rhodium‐catalyzed hydrogenation (catalyst system: [Rh(cod)_2_]OTf (cod is 1,5‐cyclooctadiene), Josiphos SL‐J505‐1) to deliver the stereoisomer *N*‐((1*S*,2*S*)‐2‐(2,4‐dichlorophenyl)cyclobutyl)acetamide in excellent yield.^36^, ^37^

Many *Cinchona*‐based quaternary salts have already been used as catalysts with varying degrees of success. To produce the active isocycloseram (5*S*,4*R*)‐stereoisomer with an ee, a cost‐efficient asymmetric technology was developed. Here, (8α,9*R*)‐(8″α,9″*R*)‐1,1″‐[9,10‐anthracanenediylbis(methylene)]bis[9‐hydroxy‐6′‐methoxy‐cinchonanium dibromide (anthracene bridge‐bound dimer)] was used as a catalyst in dichloromethane and the isocycloseram could be obtained in 82% yield (99% purity and 90% ee).^38^, ^39^

#### 2.2.4 Method IV. Enzymatic and microbial transformations

Obviously, no enzymatic and microbial transformation methods were used for the chiral agrochemicals of the past 6 years (2018–2023). Nevertheless, there is a wide range of large‐scale biocatalytic processes to produce (*S*)‐amino acids in technical quantities. In this context, industrially applicable reactions that enable enzymatic dissolution of racemates and asymmetric (bio)catalysis are particularly important.^40^ Commercial enzymatic solutions on an industrial scale are, for example, the acylase, amidase, hydantoinase and β‐lactamhydrolase‐mediated production of (*S*)‐amino acids such as (*S*)‐valine, (*S*)‐phenylalanine or (*S*)‐methionine.^41^ But in the future, whole‐cell bioconversion processes could be used, which show good tolerance at high loads with the starting material and illustrate the robustness and applicability of the biocatalysts.^42^

#### 2.2.5 Method V. Fermentation of natural products

Some precursors that are of interest to agrochemical agents can be produced by fermentation processes. Currently, for example, the natural products UK‐2A and pyripyropen A are now included, which are important for the development of the fungicide fenpicoxamid (see Section 5.2.1) and afidopyropen (see Section 6.1.1), are now included (Table S3).

Fenpicoxamid is a semi‐synthetic fungicide synthesized by alkylation of UK‐2A, which is manufactured by fermentation using *Streptomyces* sp. 517‐02 strain that can be obtained.^43^

The semi‐synthetic insecticide afidopyropen can be obtained from the natural product pyripyropen A, which was initially produced from the culture of *Aspergillus fumigatus* FO‐1289,^44^ which was later replaced by the strains *Penicillium griseofulvum* F1959^45^ and *Penicillium coprobium* PF1169 (Meiji Seika Pharma).^46^

### 2.3 Evaluation of stereoisomeric agrochemicals

An agrochemical containing a biologically active stereoisomer mixture can significantly complicate risk assessments, as each stereoisomer can have different physicochemical and toxicity properties.^17^, ^47^, ^48^ Therefore, the development of racemic agrochemicals requires a significant effort of regulatory documentation compared to achiral molecules, as it is necessary to evaluate all stereoisomers in environmental and toxicological studies. In 2000 the Environmental Fate and Effects Division (EFED) developed an interim approach for determining data requirements for non‐racemic mixtures of stereoisomeric agrochemicals.^49^ The data are essential to assess the risk posed to ecosystems and drinking water sources by these mixtures. This policy considers enantiomers or ‘optical isomers’ only. Some of the important requirements are described briefly.

Since enantiomers can exhibit stereoselective biological effects, data on the enriched mixture are required to assess its behaviors towards the racemic compound. A minimal data set for the enriched a.i. is required to determine whether the enriched mixture may pose a greater risk than the compound already registered. For the environmental fate evaluation, analytical chemical methods are required to assess the potential for stereoselectivity and to identify and quantify chiral transformation products in soil, water and fish tissues.^50^, ^51^ The differentiation of enantiomers in environmental media is important for the differentiation of biotransformation, accumulation or preferred sorption of enantiomers.^52^ In addition, chemical and physical characterization is required for all formulations containing a single enantiomer, racemic mixtures, or enantiomerically enriched mixtures. Because enantiomers may exhibit selective biological effects (e.g., soil and aquatic metabolism) may cause preferential degradation when compared to abiotic processes (e.g., hydrolysis and direct photolysis in water)^53^ an aerobic soil metabolism study is required as part of the minimal data set (fate data) for enantiomeric enriched mixtures. Furthermore, ecotoxicity data for the racemic and enantiomeric enriched mixture are needed to decide whether the data are similar or could lead to a significantly higher potential risk potential.

This makes it clear that understanding the variability of the enantioselectivity of chiral agrochemicals is very important for a reliable risk assessment of the a.i.s.

### 2.4 Significance of chiral agrochemicals in the past 6 years (2018–2023)

**Figure 1:**
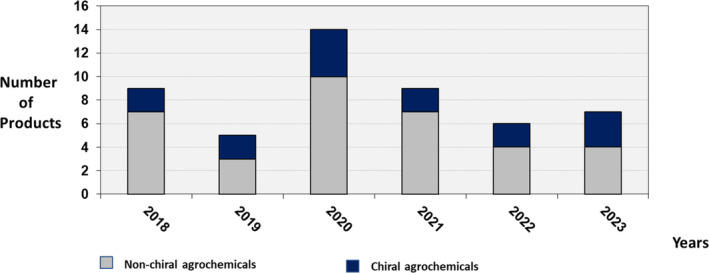
Launch of commercial non‐chiral and chiral agrochemicals in the time frame 2018–2023.

Investigation of the new agrochemicals (total 35 commercial products) used as modern crop protection pesticides, provisionally approved by the International Organization for Standardization (ISO) during the past 6 years (2018–2023, see http://www.alanwood.de) has shown that around 43% of the launched products are chiral (Fig. 1).

**Figure 2:**
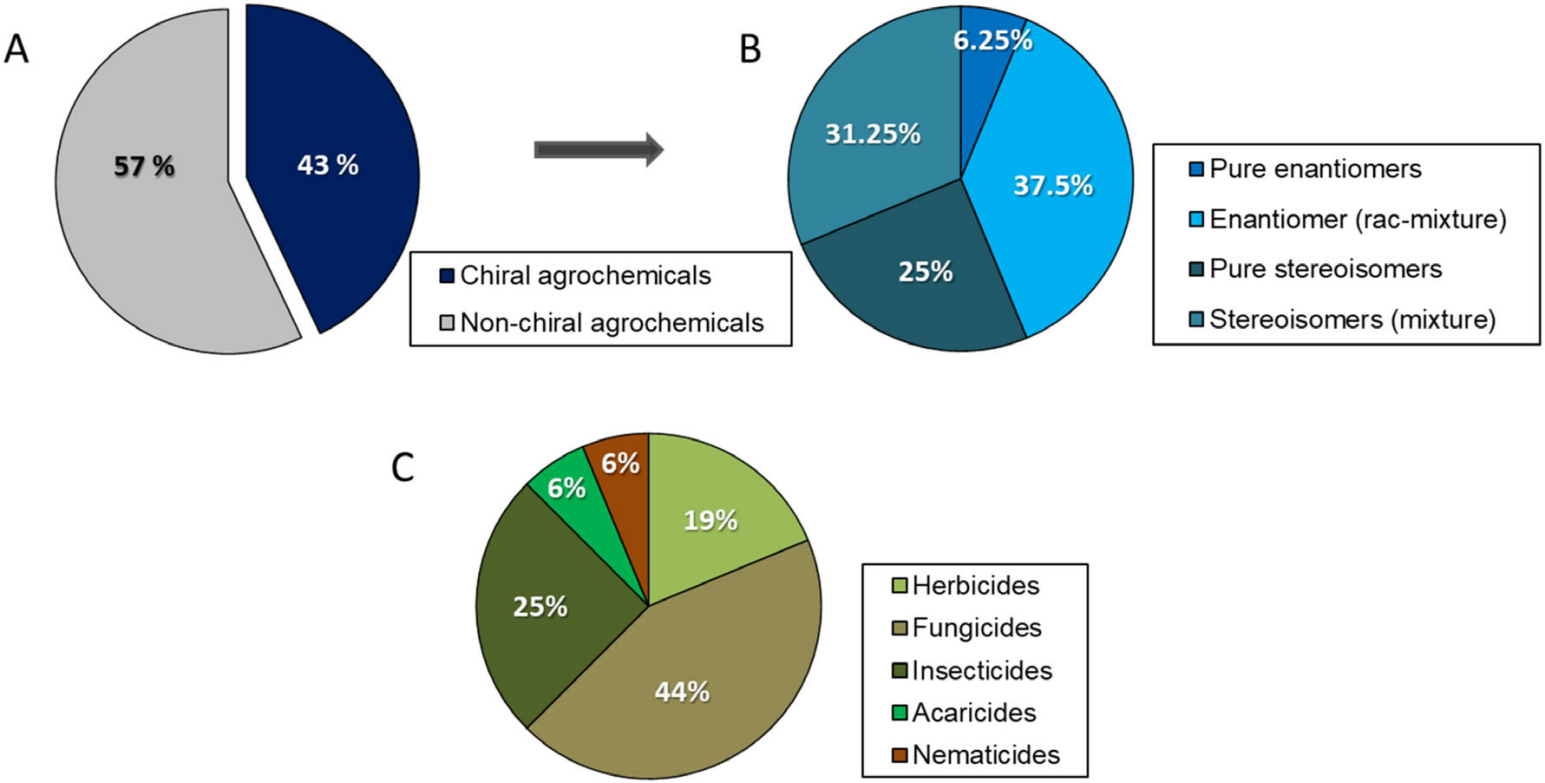
A percentage representation of known commercialized agrochemicals (2018–2023). (A) Distribution of chiral agrochemicals; (B) breakdown of chiral agrochemicals into stereochemical constitutions, and (C) breakdown of chiral agrochemicals into commercialized herbicides, fungicides, insecticides, acaricides and nematicides.

Surprisingly, the proportion of chiral compounds (three herbicides, seven fungicides, four insecticides, one acaricide, and one nematicide) is in the same order of magnitude as in the time frame from 2007 to 2017 (around 42% of the 43 launched products)^14^ and thus underlines the continuing significance of chiral agrochemicals and that the most of them consist of mixtures such as racemic mixtures of enantiomers (37.5%), mixtures of streoisomers (31.25%), followed by pure stereoisomers (25%) and enantiomers (6.25%) (Fig. 2(A)–(C)).

It becomes apparent again that the number of chiral agrochemicals with greater molecular complexity is continuously of significance.

The latest generation of chiral agrochemicals introduced to the global crop protection market in the past 6 years (2018–2023) was critically analyzed and some representative chiral pesticides with novel mechanisms of action were selected to illustrate the importance of a.i.s with stereocenters in modern agriculture.

## 3 ANALYSIS

Tables 1, 2, 3, 4 show the various classes of herbicides, fungicides, insecticides/acaricides and nematicides respectively, and is based on classification by the respective Herbicide Resistance Action Committee (HRAC; http://www. haracglobal.com),^54^ Fungicide Resistance Action Committee (FRAC; http://www.frac.info),^55^ Insecticide Resistance Action Committee (IRAC; http://www.irac-online.org)^56^ and IRAC Nematicide Mode of Action (MoA) Classification (http://irac-online.org/documents/nematicides-poster for the most complete IRAC classification, version 2.2, 11 March 2024).

**Table 1:** New agricultural chiral products launched between 2018 and 2023 as herbicides

Common name (trade name)	CAS chemical name	HRAC MoA group^†^	Target	Manufacturer	Year of launch	Comments (total number of chiral centers)	Section^‡^
Tiafenacil (Terrad'or®)	Methyl *N*‐[2‐[[2‐chloro‐5‐[3,6‐dihydro‐3‐methyl‐2,6‐dioxo‐4‐(trifluoromethyl)‐1(2*H*)‐pyrimidinyl]‐4‐fluorophenyl]thio]‐1‐oxopropyl]‐β‐alaninate	14	PPO	FarmHannong	2018	*Rac*‐mixture of (*R,S*)‐enantiomers (**1**: *RS*)	4.1.1
Cinmethylin (Luximo®)	(1*R*,2*S*,4*S*)‐*rel*‐1‐Methyl‐4‐(1‐methylethyl)‐2‐[(2‐methylphenyl)methoxy]‐7‐oxabicyclo[2.2.1]heptane	30	FAT	BASF	2020	Mixture of (1*R*,2*S*,4*S*)‐stereoisomers (**3**: 1*R*,2*S*,4*S*)	4.2.1
Tetflupyrolimet (Dodhylex^™^)	(3*S*,4*S*)‐*N*‐(2‐Fluorophenyl)‐1‐methyl‐2‐oxo‐4‐[3‐(trifluoro‐methyl)phenyl]‐3‐pyrrolidine‐carboxamide	28	DHOD	FMC	2023	(3*S*,4*S*)‐stereoisomer (**2**: 3*S*,4*S*)	4.3.1

^†^Herbicide Resistance Action Committee Mode of Action Classification 2024 (https://www.hracglobal.com).

^‡^Section number in the article.

*Note*: PPO, protoporphyrinogen‐IX‐oxidase; FAT, fatty acid thioesterase; DHOD, dihydroorotate dehydrogenase.

xxx.

**Table 2:** New agricultural chiral products launched between 2018 and 2023 as fungicides

Common name (trade name)	CAS chemical name	FRAC MoA sub‐group^†^	Target site and code	Manufacturer	Year of launch	Comments (total number of chiral centers)	Section^‡^
Mefentriflu‐conazole (Revysol®)	α‐[4‐(4‐Chlorophenoxy)‐2‐(trifluoromethyl) phenyl]‐α‐methyl‐1*H*‐1,2,4‐triazole‐1‐ethanol	G1	SBI (class I)	BASF	2019	*Rac*‐mixture of (*R,S*)‐enantiomers (**1**: *RS*)	5.4.1
Inpyrfluxam (Indiflin®)	3‐(Difluoromethyl)‐*N*‐[(3*R*)‐2,3‐dihydro‐1,1,3‐trimethyl‐1*H*‐inden‐4‐yl]‐1‐methyl‐1*H*‐pyrazole‐4‐carboxamide	C2	SDH	Sumitomo Chemical, Bayer Crop Science	2020	Pure (3*R*)‐enantiomer (**1**: *3R*)	5.1.1
Fenpicoxamid (Inatreq®)	[[4‐Methoxy‐2‐[[[(3*S*,7*R*,8*R*,9*S*)‐9‐methyl‐8‐(2‐methyl‐1‐oxoprop‐oxy)‐2,6‐dioxo‐7‐(phenylmethyl)‐1,5‐dioxonan‐3‐yl]amino] carbonyl]‐3‐pyridinyl]oxy]methyl 2‐methylpropanoate	C4	Complex III (QiI site)	Corteva Agriscience	2020	Pure (3*S*,7*R*,8*R*,9*S*)‐enantiomer (**4**: 3*S,7R*,8*R*,9*S*)	5.2.1
Fluindapyr (Onsuva®)	3‐(Bifluoromethyl)‐*N*‐(7‐fluoro‐2,3‐dihydro‐1,1,3‐trimethyl‐1*H*‐inden‐4‐yl)‐1‐methyl‐1*H*‐pyrazole‐4‐carboxamide	C2	SDH	FMC, Isagro	2021	*Rac*‐mixture of (*R,S*)‐enantiomers (**1**: *RS*)	5.1.2
Fluoxapiprolin (Xivana® Prime)	2‐[3,5‐Bis(difluoromethyl)‐1*H*‐pyrazol‐1‐yl]‐1‐[4‐[4‐[5‐[2‐chloro‐6‐[(methylsulfonyl)oxy]phenyl]‐4,5‐ ihydro‐3‐isoxazolyl]‐2‐thiazolyl]‐1‐piperidinyl]ethenone	F9	OSBP	Bayer Crop Science	2022	*Rac*‐mixture of (*R,S*)‐enantiomers.(**1**: *RS*)	5.3.1
Cyclobutrifluram (Victrato®)	*rel*‐*N*‐[(1*R*,2*R*)‐2‐(2,4‐Dichloro‐phenyl)cyclobutyl]‐2‐(trifluoro‐methyl)‐3‐pyridinecarboxamide	C2	SDH	Syngenta	2022	Mixture of (*R,S*)‐stereoisomers (**2**: 1*S*,2*S* [80–100%], 1*R*,2*R* [20–0%])	5.1.3
Florylpicoxamid (Adavelt®)	(1*S*)‐2,2‐Bis(4‐fluorophenyl)‐1‐methylethyl *N*‐[[3‐(acetyloxy)‐4‐methoxy‐2‐pyridinyl]carbonyl]‐l‐alaninate	C4	Complex III (QiI site)	Corteva Agriscience	2023	Pure (1*S*)‐enantiomer (incl. Ala) (**2**: 1S, *S*‐configurated amino acid Ala)	5.2.2

^†^Fungicide Resistance Action Committee Classification of Fungicides 2022: Fungicides sorted by mode of action (including FRAC code numbering) (http://www.frac.info).

^‡^ Section number in the article.

*Note*: SBI, sterol biosynthesis inhibitor; Complex III (quinone “outside” site), cytochrome bc1 (ubiquinone reductase) at quinone “inside” site; QiI, quinone inside inhibitor; OSBP, oxysterol binding protein; SDH, succinate dehydrogenase.

**Table 3:** New agricultural chiral products launched between 2018 and 2023 as insecticides and acaricides

Common name (Trade name)	CAS chemical name	IRAC MoA (sub)class^†^	Target	Manufacturer	Year of launch	Comments (total number of chiral centers)	Section^‡^
Afidopyropen (Inscalis®)	[(3*S*,4*R*,4a*R*,6*S*,6a*S*,12*R*,12a*S*,12b*S*)‐3‐[(Cyclopropylcarbonyl)oxy]‐1,3,4,4a,5,6,6a,12,12a,12b‐decahydro‐6,12‐dihydroxy‐4,6a, 12b‐trimethyl‐11‐oxo‐9‐(3‐pyridi‐nyl)‐2*H*,11*H*‐naphtho[2,1‐b] pyrano[3,4‐e]pyran‐4‐yl]methyl cyclopropanecarboxylate	9D	CO TRPV	BASF, Meiji Seika Pharma	2018	Pure (3*S*,4*R*,4a*R*,6*S*,6a*S*,12*R*,12a*S*,12b*S*) enantiomer (**8**: 3*S*,4*R*,4a*R*,6*S*,6a*S*,12*R*,12a*S*,12b*S*)	6.1.1
Fluxametamid (Gracia®)	4‐[5‐(3,5‐Dichlorophenyl)‐4,5‐dihydro‐5‐(trifluoromethyl)‐3‐isoxazolyl]‐*N*‐[(methoxyamino) methylene]‐2‐methylbenzamide	30	GABA	Nissan Chemical Industries	2019	*Rac*‐mixture of (*R,S*)‐enantiomers (**1**: *RS*)	6.3.1
Acynonapyr (Danyote®)	(3‐*endo*)‐3‐[2‐Propoxy‐4‐(trifluoromethyl)phenoxy]‐9‐[[5‐(trifluoromethyl)‐2‐pyridinyl]oxy]‐9‐azabicyclo[3.3.1]nonane	33	K_Ca_2	Nippon Soda	2020	Mixture of (*R*,*S*)‐stereoisomers (**3**: 1*R*,3*r*,5*S* [relative stereochemistry])	7.1.1
Isocycloseram (Plinazolin®)	4‐[5‐(3,5‐Dichloro‐4‐fluoro‐phenyl)‐4,5‐dihydro‐5‐(trifluoro‐methyl)‐3‐isoxazolyl]‐*N*‐(2‐ethyl‐3‐oxo‐4‐isoxazolidinyl)‐2‐methylbenzamide	30	GABA	Syngenta	2021	Mixture of (*R*,*S*)‐stereoisomers (**2**: 5*S*,4*R* [80–100%], 5*R*,4*R*; 5*R*,4*S*; 5*S*,4*S* [20–0%])	6.3.2
Dimpropyridaz (AxaliON™)	1‐(1,2‐Dimethylpropyl)‐*N*‐ethyl‐5‐methyl‐*N*‐4‐pyridazinyl‐1*H*‐pyrazole‐4‐carboxamide	36	CO	BASF	2023	*Rac*‐mixture of (*R,S*)‐enantiomers (**1**: RS)	6.2.1

^†^ Insecticide Resistance Action Committee Mode of Action Classification Scheme, version 11.1, January 2024 (http://www.irac‐online.org).

^‡^ Section number in the article.

*Note*: GABA, GABA‐gated chloride channel; K_Ca_2, calcium‐activated potassium channel; CO, chordotonal organ – undefined target site; CO TRPV, chordotonal organ transient receptor potential vanilloid channel.

**Table 4:** New agricultural chiral products launched between 2018 and 2023 as nematicides

Common name (trade name)	CAS chemical name	Nematicide group^†^	Target site and code	Manufacturer	Year of launch	Comments (total number of chiral centers)	Section^‡^
Cyclobutrifluram (Victrato®)	*rel*‐*N*‐[(1*R*,2*R*)‐2‐(2,4‐Dichlorophenyl)cyclobutyl]‐2‐(trifluoromethyl)‐3‐pyridinecarboxamide	N‐3	SCoQR	Syngenta	2022	Mixture of (*R,S*)‐stereoisomers (**2**: 1*S*,2*S* [80–100%], 1*R*,2*R* [20% to 0%])	8.1

^†^Insecticide Resistance Action Committee Nematicide Mode of Action Classification, https://irac‐online.org/documents/nematicides‐poster for the most complete IRAC classification, version 2.2, 11 March 2024.

^‡^ Section number in the article.

*Note*: SCoQR, succinate‐coenzyme Q reductase.

For each marketed product shown in Tables 1, 2, 3, 4, the stereochemical constitution concerning enantiomers (pure enantiomers, racemic mixtures of enantiomers, pure stereoisomers or mixture of stereoisomers) and the number of stereogenic centers are summarized, as a basis for the analysis of each of the different agrochemical areas (Fig. 2(B)).

## 4 CHIRAL HERBICIDES

Table 1 shows the HRAC MoA grouping and chemical subgrouping of the latest generation of chiral herbicides. With regard to the nine launched herbicides, three herbicides are chiral with one to three stereogenic centers, have been developed and address protoporphyrinogen‐IX‐oxidase (PPO) and two new biochemical targets or MoAs. These include fatty acid thioesterase (FAT) represented by the mixture of (1*R*,2*S*,4*S*)‐ and (*1*R,2*S*,4*R*)‐stereoisomers cinmethylin and dihydroorotate dehydrogenase (DHOD), like the new herbicide tetflupyrolimet as pure (3*S*,4*S*)‐stereoisomer. From the 19% of chiral herbicides (Fig. 2(C)), only the PPO inhibitor tiafenacil is used as racemic mixture of (*R,S*)‐enantiomers.

### 4.1 Protoporphyrinogen‐IX‐oxidase (PPO) inhibitors

The enzyme PPO (Protox, EC1.3.3.4), catalyzing the oxidation of protoporphyrinogen IX to protoporphyrin IX using molecular oxygen, is known as an important target for the discovery of modern herbicides.^57^ In plants, protoporphyrin IX is an essential substrate for the biosynthesis of chlorophyll, a key pigment for photosynthesis. The inhibition of the protox enzyme results in an accumulation of protoporphyrin IX in the cytoplasm, a strong photosensitizer of triplet oxygen, but not of the substrate via a complex process that has not yet been fully elucidated. The peroxidation process leads to the loss of membrane integrity, the pigment breakdown, and necrosis of the leaf, finally resulting in the death of the plant.

#### 4.1.1 Tiafenacil

**Figure 3:**
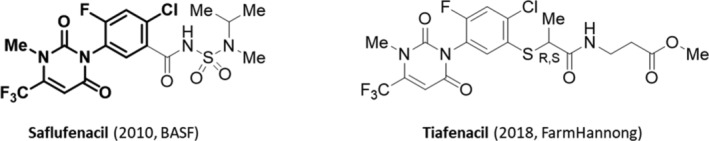
The PPO herbicides saflufenacil and tiafenacil contain the same 3,6‐dihydro‐3‐methyl‐2,6‐dioxo‐4‐(trifluoromethyl)‐1(2*H*)‐pyrimidinyl (uracil) head‐groups.

Like the PPO herbicide saflufenacil (Sharpen, 2010, BASF) the *N*‐phenyl‐imide grass herbicide tiafenacil (2018, Terrad'or, FarmHannong) contains a 3,6‐dihydro‐3‐methyl‐2,6‐dioxo‐4‐(trifluoromethyl)‐1(2*H*)‐pyrimidinyl (uracil) head group (highlighted in bold in the structure)^58^ (Fig. 3).

It is assumed the racemic thiolactic acid amide side chain of tiafenacil mimics the pyrrole ring C and the hydrophilic carboxylate moiety in protoporphyrinogen IX.^59^ Recently it has been described that various parts of tiafenacil could be modified to explore the impact on receptor binding and *in vivo* efficacy against resistant weeds.^60^

Tiafenacil is a non‐selective herbicide to both dicotyledonous and monocotyledonous weeds, such as velvetleaf (*Abutilon theophrasti*), amaranth (*Amaranthus tuberculatus*), and barnyard grass (*Echinochloa crus*‐*galli*) as well as the crops soybean, rapeseed, rice, and maize.^61^ It has been used together with glyphosate for total vegetation control in orchards and non‐till lands and is recommended for use for post emergence broadleaf and grass weed control and preplant burndown at application rates of 25–250 g a.i. ha^−1^.

### 4.2 Fatty acid thioesterase inhibitors

The FATs found in bacteria and plants have been classified into ten subfamilies.^62^ The FATs (EC 3.1.2.14), hydrolyzing the thioester bond linking acyl chains to an acyl carrier protein (ACP), thereby terminating their elongation, contribute significantly to the fatty acid (FA) content and composition of seed storage lipids.^63^ FAs synthesizing by the FA synthase complex are hydrolyzed by FATs and transported to the cytosol. The genetic manipulation of various plant FAT genes has been shown to influence FA composition.

#### 4.2.1 Cinmethylin

**Figure 4:**
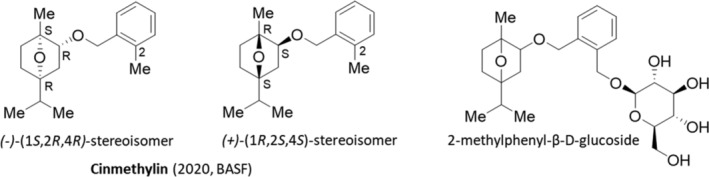
The benzyl‐ether derivative cinmethylin as a mixture of the two stereoisomers (*−*)‐(1*S*,2*R*,4*R*) and (+)‐(1*R*,2*S*,4*S*) and the *rac*‐2‐methylphenyl‐β‐d‐glucoside.

The development of cinmethylin (2020, Luximo, BASF)^64^ a benzyl‐ether derivative of the natural terpene 1,4‐cineole, described in 1981 by Shell and introduced in the market in 1989 for the use in rice, provided the first, and so far only, commercialized herbicide of this class. They also demonstrated the co‐crystallization of cinmethylin within the FAT enzyme.^65^ Cinmethylin is a mixture of the two stereoisomers (*−*)‐(1*S*,2*R*,4*R*) and (+)‐(1*R*,2*S*,4*S*) (Fig. 4), and it was listed in 2020 by the HRAC in the MoA main group Q (benzyl ethers).

Both, the optically active (*−*)‐(1*S*,2*R*,4*R*)‐cinmethylin and its enantiomer (+)‐(1*R*,2*S*,4*S*)‐cinmethylin showed similar herbicidal activity against two weed species. It has been found, that the stereochemistry (*−*)‐(1*S*,2*R*,4*R*)‐cinmethylin did not affect its herbicidal activity or spectrum.^34^ Its selective β‐d‐glycosylation of a (2‐methylphenyl)‐hydroxylated metabolite using Leloir glycosyltransferases has been studied (Fig. 4).^66^ Commercialized cinmethylin‐based products are marketed for integrated grass weed management (e.g., Luximax and Luximo) to provide control against various grasses (e.g., ryegrass and blackgrass) with developed resistances. Luximo is a soil residual herbicide for grass and broad‐leaved weed control in winter wheat, application at pre‐emergence and early post‐emergence timings. Early indications are that Luximo performs well against difficult ryegrass populations, including those populations that might be resistant to the anilide herbicide flufenacet. But cinmethylin needs to be carefully handled as part of resistance monitoring and integrated weed management (IWM) to maximize the effective longevity of this compound especially against grassweed blackgrass (*Alopecurus myosuroides*).^67^

### 4.3 Dihydroorotate dehydrogenase (DHOD) inhibitors

*De novo* pyrimidine nucleotide biosynthesis (also known as the orotate pathway) involves six enzymatic steps that lead to the formation of uridine monophosphate from carbamoyl phosphate, aspartate, and 5‐phosphoribosyl‐1‐pyrophosphate.^68^ In this context, the fourth step is catalyzed by DHOD, which enables ubiquinone‐mediated oxidation from dihydroorotate to orotate.^69^ All plant DHODs are flavoproteins located on the outer surface of the inner mitochondrial membrane, having a significantly different substrate specificity and inhibition from animal DHODs.^70^ Tetflupyrolimet (Section 4.3.1) is the first commercialized herbicide with a new mode of action in 30 years, and is an inhibitor of DHOD from the new HRAC MoA main group 28.

#### 4.3.1 Tetflupyrolimet

**Figure 5:**
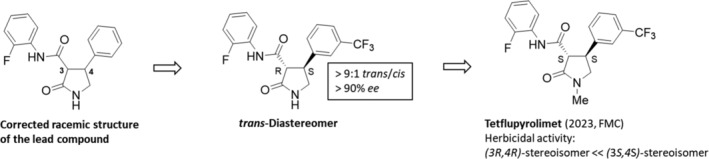
Based on the corrected racemic structure of the lead compound and followed by the *trans*‐diastereomer, the (3*S*,4*S*)‐stereosiomer of tetraflupyrolimet has been designed.

In a high‐volume sourced screening approach for miniaturized whole‐plant based glasshouse testing, the 4‐phenylpyrrolidinon‐3‐anilid provided by the vendor showed fascinating activity. Interestingly, the real structure of the supplied compound (referred to as 4‐phenylpyrrolidinone‐5‐anilide) at that time was corrected by nuclear magnetic resonance (NMR) characterization and confirmed by in‐house synthesis (Fig. 5).^71^

The target site of tetflupyrolimet (2023, Dodhylex, FMC) (Fig. 5)^72^ was found using a combination of forward genetic screening and metabolomics approaches for uncovering novel herbicide MoA from whole‐plant high‐throughput screening (HTS) efforts, and subsequently confirmed by determining the intrinsic affinities of specific analogues using biochemical methods. Structure–activity relationship (SAR) studies determined that its (3*S*,4*S*)‐enantiomer is the active form, and the counterpart had no herbicidal activity (Fig. 5). The efficacy of tetflupyrolimet was about ten‐fold greater on foxtail millet (*Setaria italica*) DHOD enzyme (*I*_50_ = 3 nm) compared to rice (*I*_50_ = 33 nm). However, its selectivity for rice is much greater than ten‐fold, suggesting that differential metabolism may also contribute to tolerance in rice. Tetflupyrolimet demonstrates excellent activity against economically important weeds such as Echinochloa, Leptochloa, and Monochoria weed species translated to the field with an excellent safety margin to rice under a variety of conditions.^71^ Thus, it provides season‐long control of important grass weeds in rice and key hard‐to‐control broadleaf weeds and sedges and can be applied to direct‐seeded rice.

### 4.4 Selected chiral development candidate herbicides

Other chiral herbicides with different mechanisms of action are currently under development. KingAgroot CropScience Co., Ltd has developed the four herbicides flufenoximacil (ISO‐proposed, June 2022), fluchloraminopyr (ISO‐proposed, June 2022), flusulfinam (ISO‐proposed, August 2022) and cinflubrolin (ISO‐proposed, January 2024) (Supporting Information Fig. S1).

Recently, the registration of the three herbicides in China has reached the final stage, scheduled for market launch in China in 2023–2024.

The pyridyloxycarboxylic acid herbicide flufenoximacil (trade name Kuairufeng)^73^ is a new fast‐acting PPO inhibitor with a wide herbicidal spectrum against numerous grass weeds. It acts quickly, demonstrating exceptional effectiveness against goosegrass (*Eleusine indica*) and horseweed herb (*Erigeron canadensis*).

The non‐selective proherbicide^74^ fluchloraminopyr‐tefuryl the ester of fluchloraminopyr^75^ has a broad herbicidal spectrum for effectively controlling a large variety of glyphosate‐resistant and tolerant weeds such as *Erigeron canadensis*, *Eleusine indica*, dayflower (*Commelina communis*), field bindweed (*Convolvulus arvensis*), and rice cutgrass (*Lecrsia oryzoides*). It also is effective against the tough shrubs and vines in woodland and non‐arable land.

The racemic enantiomer of the 4‐hydroxy‐phenylpyrovate dioxygenase (4‐HPPD) inhibitor flusulfinam (mixture of 80–100% of the 3‐(*R*)‐enantiomer and 0–20% of the 3‐(*S*)‐enantiomer; Trade name Daopurui®)^76^ shows effective control of parts of broadleaf and sedge weeds (*Cyperus difformis*) as well as critical weeds in rice fields, such as resistant field grass (*Echinochloa crus*‐*galli*), crabgrass (*Digitaria sanguinalis*), mole plant seeds (*Euphorbia lathyrus*), which are resistant to acetolactate synthase (ALS) and acetyl‐CoA‐carboxylase (ACCase) inhibitors. Flusulfinam is very safe for the use of rice, this also applies in an analogous way to the varieties Japonica rice and Indica rice. The a.i. is active on the stem, leaf and soil of the rice plant. Studies of the enantioselective bioactivity, toxicity and degradation of flusulfinam have shown that its (*R*)‐enantiomer is less toxic and at the same time more active for the tested species. The half‐life (degradation time; DT_50_) of its (*R*)‐ and (*S*)‐enantiomers in rice (*Oryza sativa* L.) is 5.50 and 5.06 days (*P* < 0.05), supporting the preferential degradation of the (*S*)‐enantiomer throughout the total rice plant.^77^ It is assumed, that the special up‐regulation of the *lipid transfer protein*‐*2* and *carboxylesterases15* genes could explain the preferential transport within the rice plant and degradation of the (*S*)‐enantiomer.

The halogen‐containing FAT inhibitor cinflubrolin (ISO‐proposed, January 2024)^78^ is a derivative from structural surroundings of the herbicide cinmethylin (see Section 4.2.1). Instead of the 2‐[(2‐methylphenyl)methoxy] fragment in cinmethylin, a 2‐[(2‐bromo‐6‐fluoro‐phenyl)methoxy] fragment is present in cyflumetyline (mixture ≥ 50% of the (1*S*,2*R*,4*R*)‐stereoisomer and ≤ 50% of the (1*R*,2*S*,4*S*)‐stereoisomer).

The non‐selective proherbicide icafolin‐methyl^79^ (ISO‐proposed, January 2022) is a novel, highly effective herbicide against the most relevant competitive weeds in cold and warm season cropping systems at low application rates, including resistant black‐grass and rye‐grass biotypes. It demonstrates activity against several invasive plants, such as bittergrass (*Cardamine hirsute*) and crow's foot grass (*Eleusine indica*) with strong post‐emergence and some residual activity, specifically after foliar application. Icafolin‐methyl (mixture of 40–70% of the (2*R*,4*R*)‐isomer and 60–30% of the (2*S*,4*S*)‐isomer), which hydrolyses in planta to the carboxylic acid icafolin (ISO‐proposed, January 2022), belongs to the chemical class isoxazolin carboxamide and acts through plant‐specific inhibition of tubulin polymerization probably by binding to β‐tubulins.

## 5 FUNGICIDES

Table 2 outlines the FRAC MoA grouping of the latest generation of chiral fungicides. Accordingly, of the 11 fungicides launched from 2018 to 2023, seven products (around 44%) are chiral, mostly with one or more stereogenic centers. They mainly target the respiratory chain, addressing the biochemical target succinate dehydrogenase (SDH),^80^ cytochrome bc_1_ (ubiquinone reductase) at quinone inside inhibitor (QiI), sterol biosynthesis inhibitor (SBI) (class I), and oxysterol binding protein (OSBP).

### 5.1 Fungicidal succinate dehydrogenase (SDH) inhibitors

**Figure 6:**
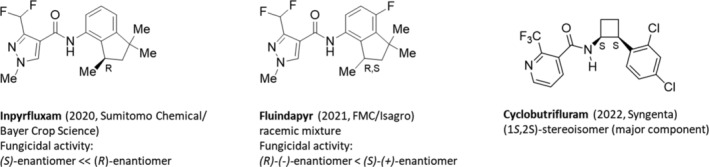
Chemical structures of the SDH inhibitors inpyrfluxam as (*R*)‐enantiomer, fluindapyr as racemic mixture and cyclobutriflam as (1*S*,2*S*)‐stereoisomer (major component).

Over the past decade, there has been a significant increase in the number of chiral fungicides targeting SDH (complex II). Currently, 24 commercialized SDH inhibitor fungicides are described in the FRAC MoA poster (market and development products; http://www.frac.info), which was updated in March 2024. About 42% of them have one or more stereogenic centers in the molecule, which contribute to their broad spectrum of activity against plant pathogens. The chemical sub‐group pyrazol‐4‐carboxamides consists of ten members, eight of which have stereogenic centers in the molecule and seven of them are racemates. Only the latest fungicide inpyrfluxam is marketed as a pure (*R*)‐enantiomer (Fig. 6).

Today, the 3‐difluoromethyl‐pyrazol‐4‐ylcarboxylic acid is produced very economically in an industrial manufacturing process. The acid is then combined with the respective chiral amine component via an ‘amide linker’.^58^, ^81^

#### 5.1.1 Inpyrfluxam

As pure (*R*)‐enantiomer, inpyrfluxam (2020, Indiflin, Sumitomo Chemical/Bayer AG, Crop Science; optical purity > 99%) (Fig. 6), is more active as the corresponding racemic mixture. The (*S*)‐enantiomer is nearly inactive.^82^ The broad spectrum and systemic SDH fungicide inpyrfluxam is highly effective against basidiomycetes and ascomycetes, such as brown patch (*Rhizoctonia solani*) and apple scab (*Venturia inaequalis*).^82^ It shows efficacy against Asian soybean rust (*Phakopsora pachyrhizi*), an important disease protection for the soybean market in Brazil. Because of its systemic efficacy inpyrfluxam is effective in controlling Asian soybean rust (ASR), even in fields with putative SDH inhibitor‐resistant populations, suggesting this compound as a solution for innovative soybean protection. In addition, inpyrfluxam provides control of peanut foliar and soil‐borne diseases found in southwest peanut production.^83^

Studies have shown, that during photodegradation of inpyrfluxam in water and nitrate aqueous solution no isomerization occurred at the 3′‐position of its indane ring.^84^

#### 5.1.2 Fluindapyr

The racemic fluindapyr (2021, Onsuva, FMC/Isagro) (Fig. 6) contains a racemic 7‐fluoro‐1,1,3‐trimethyl‐indan‐4‐yl moiety and can be used for preventive and curative control of fungal diseases in various key crops such as cereals, soybeans, corn, oilseed rape, fruits and vegetables, tree nuts and peanuts. After isolation of the enantiomers (see Section 2.2, Table S1), it has been shown that the fluindapyr (*R*)‐(*−*)‐enantiomers is degraded faster than its (*S*)‐(+)‐enantiomer in the rice soil, while the (*S*)‐(+)‐enantiomer against brown spots (*Rhizoctonia solani*) is more active than the (*R*)‐(−)‐enantiomer.^27^ In addition, both enantiomers show different enantioselective degradation behavior in different plant species. For example, the (*R*)‐(−)‐enantiomer is preferentially degraded in tomato leaves, while the (*S*)‐(+)‐enantiomer is preferentially degraded in cucumber leaves. Possibly the enantioselectivity of degradation is controlled by different enzyme systems.^85^

#### 5.1.3 Cyclobutrifluram

Cyclobutrifluram (2022, Victrato, Syngenta) (Fig. 6),^36^, ^37^ a mixture comprising 80–100% of the (1*S*,2*S*)‐stereoisomer and 20% to 0% of the (1*R*,2*R*)‐stereoisomer is a member of the new SDH inhibitor sub‐group phenyl‐cyclobutyl pyridineamides. Its structure was inspired by the non‐chiral fluopyram (2007, Luna, Bayer Crop Science),^58^, ^86^ which contains a [–CH_2_–CH_2_–]‐ethylene linker between the amide group and the heterocycle. The catalytic asymmetric synthesis of the cyclobutrifluram (1*S*,2*S*)‐stereoisomer is described in Section 2.2 (see Table S2). Cyclobutrifluram products for soil application are marketed as either Vaniva® 45 SC or Evidis, with the target crops being potatoes, tomatoes, bananas and sugar cane. Victrato® 50 FS is used for seed treatment, which focuses on nematodes and important soil‐borne fungal diseases and increases the quality and yield of many crops such as soybeans, corn, cereals, cotton and rice. Safe for beneficial insects, pollinators and the soil microbiome, the product enables no‐till and conservation tillage by protecting roots. Victrato® contains Tymirium® technology, a high‐performance, low‐dose drug innovation.

### 5.2 Fungicidal quinone inside inhibitors (QiIs)

**Figure 7:**
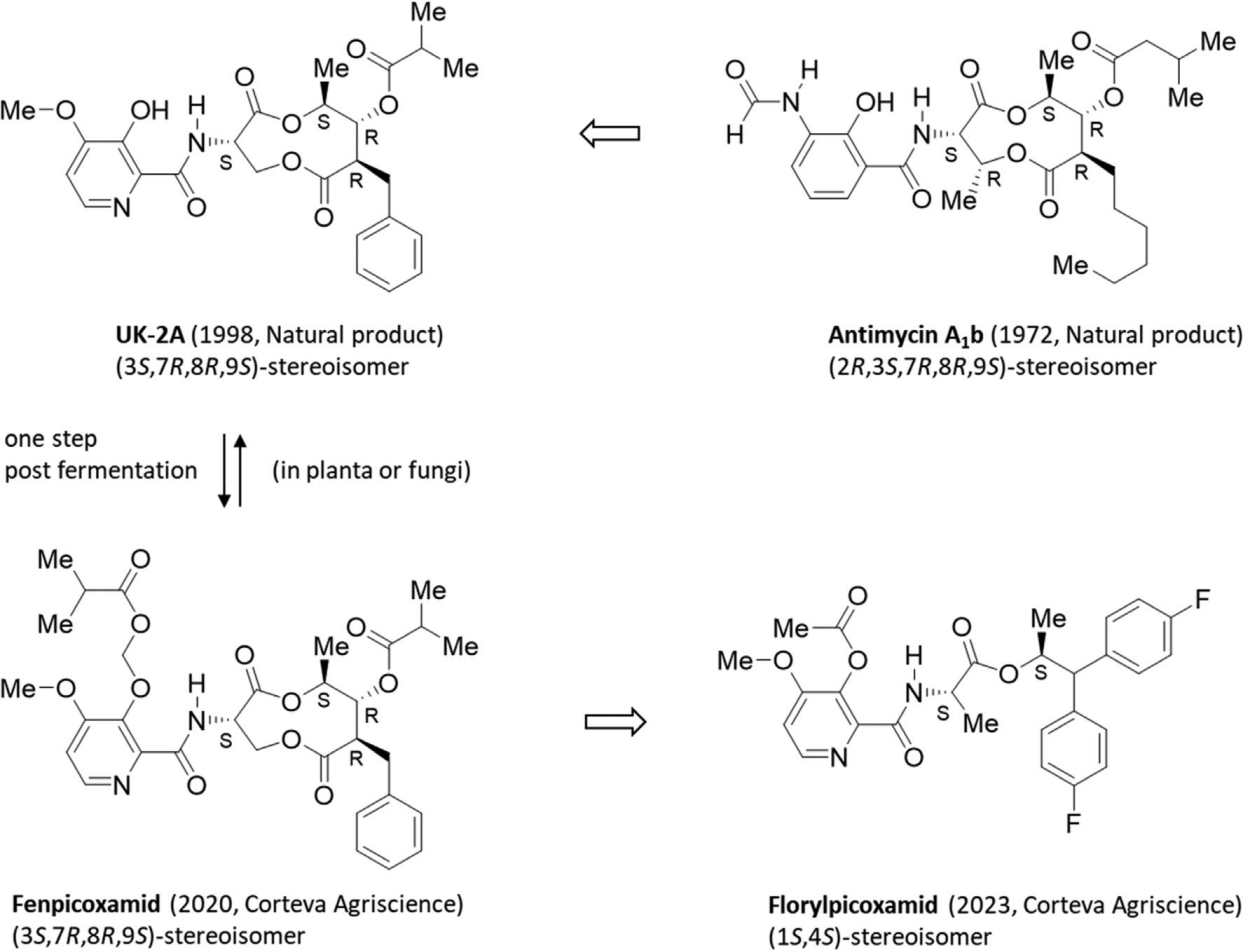
Synthesis of the fungicide fenpicoxamid based on the natural products antimycin A_1_b, UK‐2A and its subsequent structural simplification, which resulted in the fungicide florylpicoxamid.

The natural product antimycin A_1_b (secondary metabolite produced by *Streptomyces bacteria*),^87^ an (2*R*,3*S*,7*R*,8*R*,9*S*)‐stereoisomer containing five stereocenters in a nine‐membered 4,9‐dioxo‐1,5‐dioxononane ring system, has inhibitory activity at the cellular target respiratory chain complex III cytochrome bc1 (ubiquinone reductase) at quinone “inside” site.^88^ Currently, four synthetic QiI fungicides are described in the MoA poster (market and development products; http://www.frac.info), which was updated in March 2024. Only two of them, the fungicides fenpicoxamid and florylpicoxamid, belong to the chemical class of picolinamides have four and two stereocenters, respectively, in the molecule (Fig. 7).

So far, the detailed binding mode of picolinamide fungicides is still unknown. In a recent study, antimycin‐A and UK‐2A were selected to investigate the binding mode of picolinamide inhibitors with four protonation states in the quinone “inside” site by integrating molecular dynamics simulation, molecular docking, and molecular‐mechanical generalized born surface area (MM/GBSA) calculations.^89^

#### 5.2.1 Fenpicoxamid

The semi‐synthetic acyloxymethyl ether pro‐fungicide fenpicoxamid (2020, Inatreq, Corteva) (Fig. 7)^90^ based on the natural product UK‐2A an antifungal, natural metabolite isolated from the fermentation broths of the actinomycete *Streptomyces* sp. 517‐02 (see Section 2.2, Table S3)^91^ extracts and its total synthesis.^92^ It was identified that UK‐2A can be modified at the benzyl position on the macrocycle, providing opportunities for optimization of its physical properties while retaining its strong intrinsic and antifungal activity.^93^ The substitution of the isobutyryl ester group of UK‐2A results in strong antifungal activity against wheat leaf spot disease (*Zymoseptoria tritici*) and other fungi.^94^ It has been found, that fenpicoxamid can be prepared by a one‐step O‐alkylation of the picolinamide hydroxy group of UK‐2A. It works as pro‐fungicide, since it is converted to the natural product UK‐2A in crops. Due to the novel target site for the cereals market, *Z. tritici* strains resistant to strobilurine and/or azole fungicides are not cross‐resistant to fenpicoxamid. The chemistry of picolinamides resulted in a novel biochemical MoA for the cereal fungicide market involving inhibition of mitochondrial complex III via binding to the quinone "inside" site of the respiratory cytochrome bc1 complex^95^ rather than to the quinone "outside" site targeted by the strobilurin class of fungicides. Therefore, no target‐site‐based cross‐resistance to strobilurin fungicides can be anticipated.

#### 5.2.2 Florylpicoxamid

It has been assumed that the 4‐methoxy‐3‐hydroxy‐picolinamide head group is the main pharmacophore. Therefore, further design has been focused on the replacement of the macrocyclic bis‐lactone amine tail with a more simplified amine moiety, which could guarantee the chemical space between the complex UK‐2A tail and methylamine. The enormous size of this chemical space was a challenge to find an effective replacement for the UK‐2A tail. Instead of randomness, a function‐oriented synthesis strategy^96^ was used to identify the structural elements present in the UK‐2A tail that are important for *in vivo* fungicidal efficacy to obtain a simplified structure.

The molecule design of the (1*S*,2*S*)‐diastereomer florylpicoxamid (2023, Adavelt, Corteva) (Fig. 7),^97^, ^98^, ^99^ focused on retaining structural features (considering the stereochemistry) to binding at the ubiquinone quinone inside target site of mitochondrial complex III of the respiratory chain of the ‘sugar‐fungus’ *Saccharomyces cerevisiae*.^95^ By deconstruction of the macrocyclic ring UK‐2A via total synthesis revealed key structural features important for the *in vivo* control of fungal diseases. The most efficacious stereoisomer of the active ingredient is manufactured from the natural lactic acid as (*S*)‐lactide and the amino acid (*S*)‐alanine (see Section 2.2, Method II). The trisubstituted pyridine can be prepared from furfural, a renewable feedstock.^100^ Florylpicoxamid demonstrates control of wheat leaf blotch (*Z. tritici*) fungal diseases, which are translated to control of other ascomycete pathogens such as tomato early blight (*Alternaria solani*), sugar beet leaf spot (*Cercospora beticola*), cucumber anthracnose (*Colletotrichum orbiculare*), grape powdery mildew (*Uncinula necator*), rice blast (*Pyricularia oryzae*), and barley scald (*Rhynchosporium secalis*).

Florylpicoxamid provides farmers with an innovative solution to maintain increased productivity and quality of numerous crops and provides an option for fungicide resistance management.^101^

### 5.3 Oxysterol binding protein (OSBP) inhibitors

The molecular target of OSBP inhibitors is the OSBP, a member of the OSBP‐related protein (ORP) family of lipid transfer proteins (LTPs).^102^ They are a family of sterol and phosphoinositide binding and transfer proteins in eukaryotes, conserved from yeast to humans. OSBP localizes to endoplasmic reticulum‐Golgi contact sites, where it transports cholesterol and phosphatidylinositol‐4‐phosphate and activates lipid transport and biosynthetic activities.^103^ OSBP fungicides are effective against oomycete fungi and used for the control of potato late blight (*Phytophthora infestans*) and downy mildews (*Plasmopara viticola*) of numerous crop plants. OSBP inhibitors inhibit an OSBP homologue. Inhibiting OSBP may disrupt processes in the fungal cell, such as signaling, maintaining cell membranes, and the formation of more complex lipids that are essential for the cell to survive.

**Figure 8:**
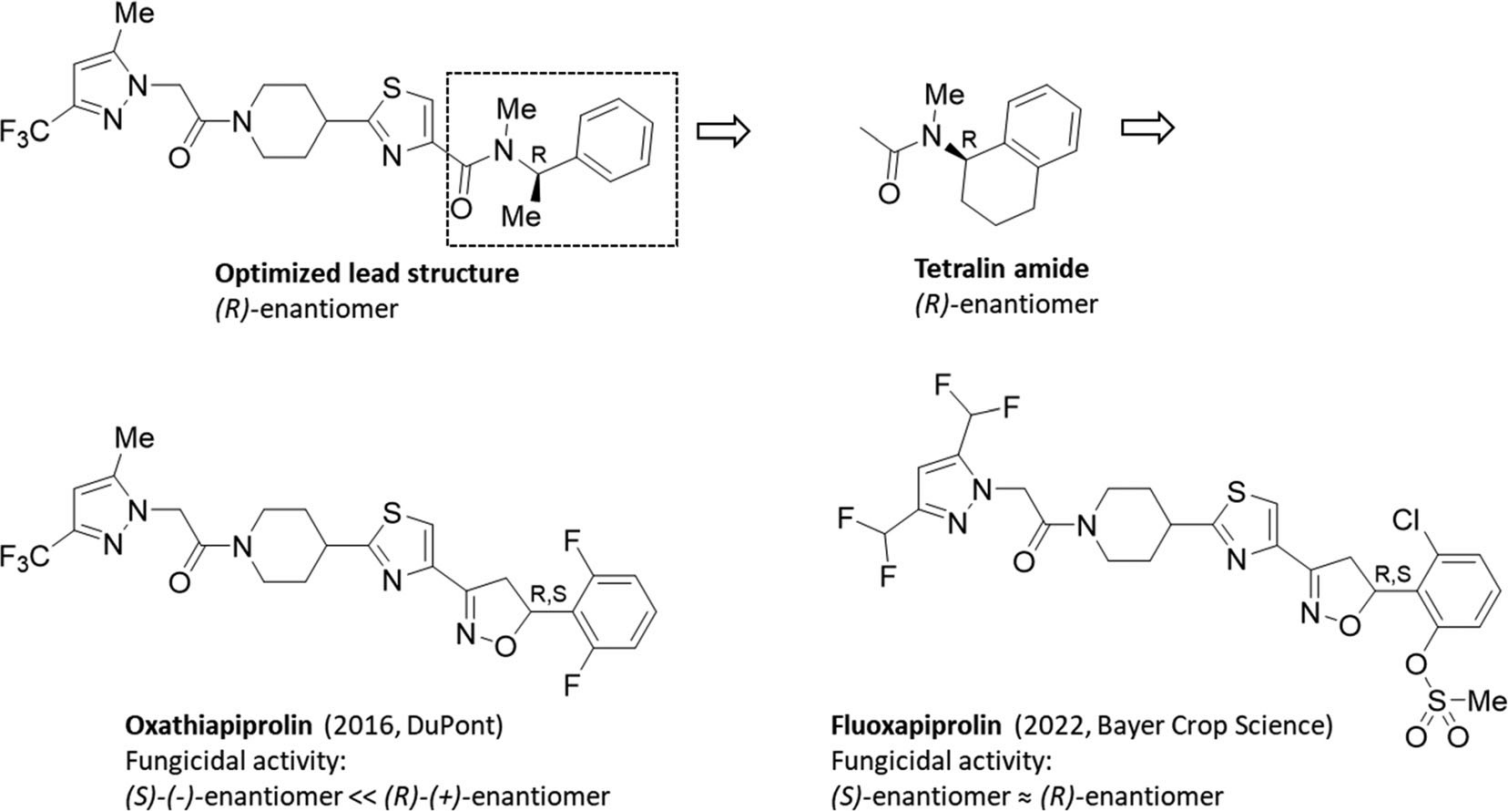
Based on HTS results and the optimization strategy, the racemic fungicide oxathiapiprolin was developed, which subsequently inspired the discovery of the racemic fluoxapiprolin.

The racemic oxathiapiprolin (2016, Zorvec, DuPont) (Fig. 8)^58^, ^104^ is the first member of the class of the piperidinyl‐thiazole isoxazoline fungicides, exerting an excellent preventive, curative and residual efficacy against diseases in grapes, potatoes and vegetables at low use rates. Oxathiapiprolin is a chiral fungicide consisting of two enantiomers whose fungicidal activity and degradation in the environment have been studied.

For example, its *R*‐(*−*)‐enantiomer is 2.49*–*13.30 times more fungicidal than the *S*‐(+)‐enantiomer in a test against six types of pathogens, while the degradation rate of the *R‐*(*−*)‐enantiomer was slightly faster than that of its (*S*)‐(+)‐enantiomer after application to tomatoes and peppers.^105^

#### 5.3.1 Fluoxapiprolin

The structurally related and second member of the class of the piperidinyl‐thiazole isoxazoline fungicides is the racemic fluoxapiprolin (2022, Xivana Prime, Bayer Crop Science) (Fig. 8)^106^ containing a 3,5‐bis(difluoromethyl)‐1*H*‐pyrazol‐1‐yl moiety and a 2‐chloro‐6‐[(methylsulfonyl)oxy]phenyl group in its total molecule. The respective (*R*)‐ and (*S*)‐enantiomers showed almost the same level of inhibitory activity to plant pathogenic oomycetes. Fluoxapiprolin is a foliar fungicide for reliable *Plasmopara viticola* control in grapes with high efficacy and very low dose rates. Its long‐lasting efficacy (10–21 days) allows longer spray intervals and flexible application, even under rainy conditions. Fluoxapiprolin shows a wide application range throughout the season, including good compatibility with other tank mix partners for control of additional diseases. In addition, it demonstrates an excellent safety profile for pollinators and beneficial insects at application during the flowering period. Since fluoxapiprolin exhibits the same OSBP homologue inhibition as oxathiapiprolin, positive cross‐resistance could be expected (see https://www.frac.info/frac-teams/working-groups/osbpi-fungicides/information), which has been investigated therefore for *Phytophthora infestans*.^107^

According to the general FRAC recommendations for OSBP inhibitor fungicides of the OSBP inhibitor working group from January 2024, the resistance risk is assumed to be medium to high (single site inhibitor) and resistance management is recommended. For instance: (a) the application of OSBP inhibitors is only preventative and in mixtures with effective fungicides from different cross‐resistance groups, (b) the mixture partner should give effective control of the target disease(s) at the rate and interval selected, and (c) foliar exposure to OSBP inhibitor products should not exceed 33% of the total period of protection needed per crop.

### 5.4 Sterol biosynthesis inhibitors (class I)

Sterol biosynthesis in membrane inhibitors, in particular sterol‐C_14_‐demethylase inhibitors in sterol biosynthesis (*erg11/cyp51*) [DMI‐fungicides (SBI: class I) and number in parentheses such as triazoles (26), piperazines (1), pyridines (2), pyrimidines (2), imidazoles (5) and triazolinthiones (1); according to FRAC = G1] still belong to the most important broad‐spectrum fungicides and around 78% of them contain chiral centers. None are launched as enantiomerically enriched or pure enantiomers, because both effects that can be attributed to each stereoisomer (fungicidal and plant growth regulation efficacy) are desirable.^7^ All DMIs block a specific cytochrome P_450_‐enzyme, responsible for the oxidative C_14_‐demethylation of the intermediate C_24_‐methylene dihydrolanosterol in the sterol biosynthesis pathway of agricultural pathogens.^108^

#### 5.4.1 Mefentrifluconazole

**Figure 9:**
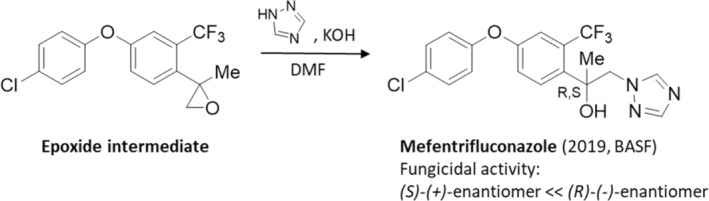
Synthetic pathway of the racemic mefentrifuconazole, a fungicidal sterol biosynthesis inhibitor.

The racemic fungicide mefentrifluconazole (2019, Revysol, BASF) (Fig. 9)^109^ can be prepared from the epoxide intermediate, obtainable by using 1‐(4‐(4‐chlorophenoxy)‐2‐(trifluoromethyl)phenyl)ethan‐1‐one via Corey–Chaykovsky reaction after ring opening with 1,2,4‐triazole.^58^, ^110^ It provides protection to many cereals and legume vegetables, including maize, soybean, and sugar beet. Mefentrifluconazole has substantial antifungal activity against a wide range of pathogenic fungi such as *Septoria tritici* blotch (STB) of wheat, brown rot of stone fruits (*Monilinia fructicola*), and gray mold‐rot (*Botrytis cinerea*).

It has been found that the (*R*)‐(−)‐enantiomer of mefentrifluconazole is 11–113 times higher bioactive against numerous phytopathogens (*Alternaria solani*, *B. cinerea*, *Rhizoctonia solani*, *Colletotrichum gloeosporioides*, and *Aspergillus fumigatus*) than its (*S*)‐(+)‐enantiomer (see Section 2.2, Table S1).^111^ Recently, the stereoselective activity of the two enantiomers against *Fusarium verticillioides* and their differences in mycotoxin fumonisin biosynthesis has been investigated by determining the inhibition of the strain, hyphae and conidia. It has been shown that the inhibition of the strain and conidia by the (*R*)‐(−)‐enantiomer was greater than that of the *S*‐(+)‐enantiomer.^112^ If the mechanism of selective bioactivity against *F. verticillioides* and fumonisin biosynthesis are assessed for the enantiomers of mefentrifluconazole, then the (*R*)‐(−)‐enantiomer shows a stronger binding to proteins than *S*‐(+)‐enantiomers.^112^ In addition, based on AlphaFold2 (AF2) modeling and molecular docking, both enantiomers demonstrate different binding modes with key target proteins in pathogens and zebrafish, which may be the main cause for their stereoselective differences in bioactivity and biotoxicity.^113^

### 5.5 Selected chiral development candidate fungicides

Two more chiral fungicides with the already mentioned MoAs are currently in development by Corteva Agriscience. The pro‐fungicide metarylpicoxamid (Haviza; ISO‐proposed, March 2021) (Fig. S2),^114^, ^115^ the third generation of picolinamide fungicides acting as QiIs (see Section 5.2). It has the potential for strong protective and curative control of ASR, caused by the biotrophic pathogen *Phakopsora pychyrhizi*, continues to be a devasting disease in soybeans.

It is planned, that Haviza will be offered in mixtures primarily with the strobilurine fungicide picoxystrobin (2010, Onmira active, originator Zeneca and current owner DuPont), providing market‐leading control of ASR and expanding control to key late‐season diseases. The triazolinthione fluoxytioconazole (ISO‐proposed, March 2021) (Fig. S2),^115^, ^116^ discovered by Viamet Pharmaceuticals (now Mycovia Pharmaceuticals) and registered by Corteva Agriscience is a new sterol‐C_14_‐demethylase inhibitor. After prothioconazole (2024, Proline, Bayer Crop Science),^108^ is the second member of the triazolinthione sub‐group according to the FRAC MoA classification. Fluoxytioconazole controls a range of significant plant diseases, including Septoria and rust on cereals and sugar beet, and the leaf‐spot disease black sigatoka on bananas.

## 6 INSECTICIDES

Table 3 lists the IRAC MoA grouping of the latest generation of chiral insecticides. Accordingly, of the 12 insecticides launched on the market in the period from 2018 to 2023, four products (around 33%) are chiral, mostly with one or more stereogenic centers. These insecticides mainly show efficacy at the TRPV (transient receptor potential vanilloid) channel of the chordotonal organ as well as its undefined target site and the γ‐aminobutyric acid (GABA)‐controlled chloride channel.

### 6.1 Chordotonal organ TRPV channel modulators

The chordotonal organ TRPV channel modulators bind to and disrupt the gating of Nanchung (Nan) and Inactive (Iav) form complexes in chrodotonal stretch receptor organs, which are critical for hearing, gravity, balance, acceleration, proprioception, and kinesthesia. This disrupts feeding and other behaviors in target insects.

#### 6.1.1 Afidopyropen

**Figure 10:**
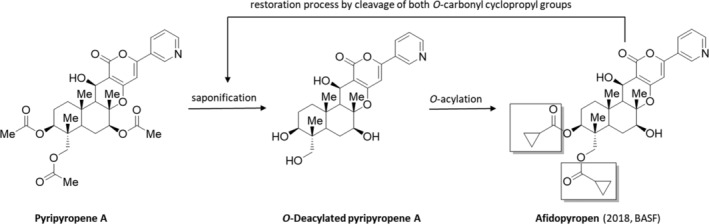
Manufacturing process of afidopyropen based on the natural product pyripyropene A.

Afidopyropen (2018, Inscalis, BASF/Meiji Seika Pharma) (Fig. 10)^117^, ^118^ is a potent and specific TRPV channel modulator, which can be over‐stimulated and eventually silenced by the two commercial insecticides such as pymetrozine and pyrifluquinazone. The natural, fungal fermentation process for afidopyropen uses a recombinant strain of the amorph filamentous fungus species *Penicillium coprobium* to manufacture the pyropen skeletal (see Section 2.2, Table S3), thereby provided eight stereogenic centers and four oxygen functionalities with high selectivity.^119^

By this procedure afidopyropen can be prepared in an unselective two step manufacturing process. In the first step pyripyropene A is O‐deacetylated under basic conditions (saponification) without any racemization of the eight stereogenic centers in the natural product. After O‐acylation of two hydroxyl groups with cyclopropane carbonyl chloride, mainly afidopyropen is formed. The tri‐O‐acylated by‐product can be easily recycled into O‐deacylated pyripyropene A through an efficient combination of saponification and recovery process.^119^, ^120^

Afidopyropen is a foliar insecticide that acts quickly by disrupting feeding, which leads to reduced virus transmission in vegetable crops. It shows good translaminar distribution but is not completely systemic. Afidopyropen enables effective control of stinging and sucking insect pests such as aphids, whiteflies,^121^ various scale insects, cicadas, certain psyllids (e.g., *Asian citrus psyllides*) and dandruff, including those that have already developed resistance to other insecticides. It is registered by the United States Environmental Protection Agency (US EPA) for use in soybeans, tubers and corms, *Brassica* head and stem, fruiting and leafy vegetables, cucurbits, potatoes, pome fruit, stone fruit, tree nuts, and ornamentals. Afidopyropen has been classified as the first member of the novel class of pyropenes of MoA sub‐group 9D according to the IRAC MoA classification.

### 6.2 Cordotonal organ modulators – undefined target site

The cordotonal organ modulators with undefined target site (IRAC MoA main group 36) interfere with the function of the chordotonal distension receptor organs, which are critical for hearing, gravity, balance, acceleration, proprioception, and kinesthesia. This hinders feeding and other behaviors in target insects. The pyridazine pyrazolecarboxamides (PPCs) exemplified by dimpropyridaz (Section 6.2.1) of this group act in a different place than chordotonal organ TRPV channel modulators (IRAC MoA main group 9; see Section 6.1.1, afidopyropen from MoA sub‐group 9D) and chordotonal organ nicotinamidase inhibitors (IRAC MoA main group 29) and do not affect TRPV channels or nicotinamidase.

#### 6.2.1 Dimpropyridaz

**Figure 11:**
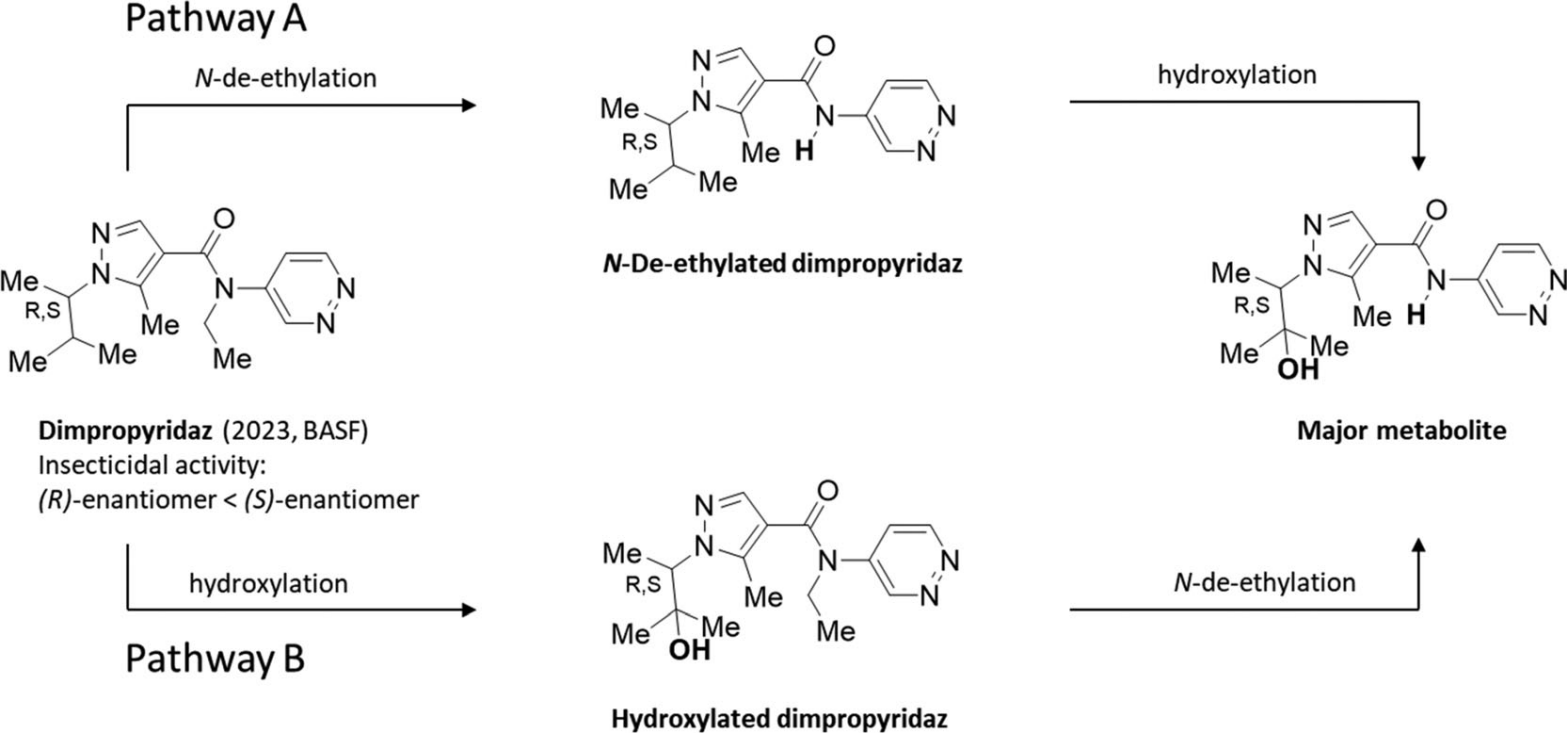
Proposed metabolic pathways of dimpropyridaz in green peach aphid. Dimpropyridaz is either first transformed to the N‐de‐ethylated dimpropyridaz, which can be further hydroxylated to the major metabolite (pathway A), or it is first transformed to the hydroxylated dimpropyridaz followed by N‐de‐ethylation resulting in the major metabolite (pathway B) (according to Spalthoff *et al*.^124^).

The racemic dimpropyridaz (2023, Axalion, BASF) (Fig. 11),^122^ is a pro‐insecticides that is metabolized in target insects by N‐de‐ethylation to its active form, which acts directly on chordotonal organs.^123^, ^124^ It was found that dimpropyridaz is either first N‐de‐ethylated at the amide, forming N‐de‐ethylated dimpropyridaz, which can be further hydroxylated at the iso‐propyl fragment resulting in the major metabolite, or it forms the hydroxylated dimpropyridaz as intermediate which after N‐de‐ethylation forms the major metabolite.

Independent of dosing, the majority of dimpropyridaz can be metabolized to the major metabolite (above 90% of the sum of the determined metabolites) and N‐de‐ethylated dimpropyridaz as well as the hydroxylated dimpropyridaz as minor metabolites in aphids plus honeydew samples.^124^

The insecticide Axalion controls a broad spectrum of problematic piercing and sucking pests (e.g., use rates for aphids range from 24 to 60 g a.i. ha^−1^), including whiteflies (life stage control: adults, eggs, larva second to fourth instar).^122^ Its uses include fruits, vegetables, soybeans, legumes, cotton, cereals, beets, oilseed rape and ornamentals. By using foliar spray, drench and drip applications, the translaminar and inherent systemic properties of Axalion provide long‐lasting residual control and make possible growers a wide window of application timing during early to late growth stages. This includes late growth stage spraying. Finally, the product Efficon® (soluble liquid, SL), containing 120 g l^−1^ dimpropyridaz (Axalion active), has been approved for the control of silverleaf and glasshouse whiteflies in cotton, cucurbit, and fruiting vegetable crops. It is also active against green peach aphids and cabbage aphids in brassica and leafy vegetable crops, and cotton (or melon) aphids in cotton and cucurbit crops. In addition, a reduction of beet yellow virus (BYV) transmission up to 96% with a use rate of 44 g a.i. ha^−1^ has been described.^125^ So far, no cross‐resistance has been found, therefore it will be an effective tool for insect resistance management (IRM) strategy.

### 6.3 GABA‐gated chloride channel allosteric modulators

**Figure 12:**
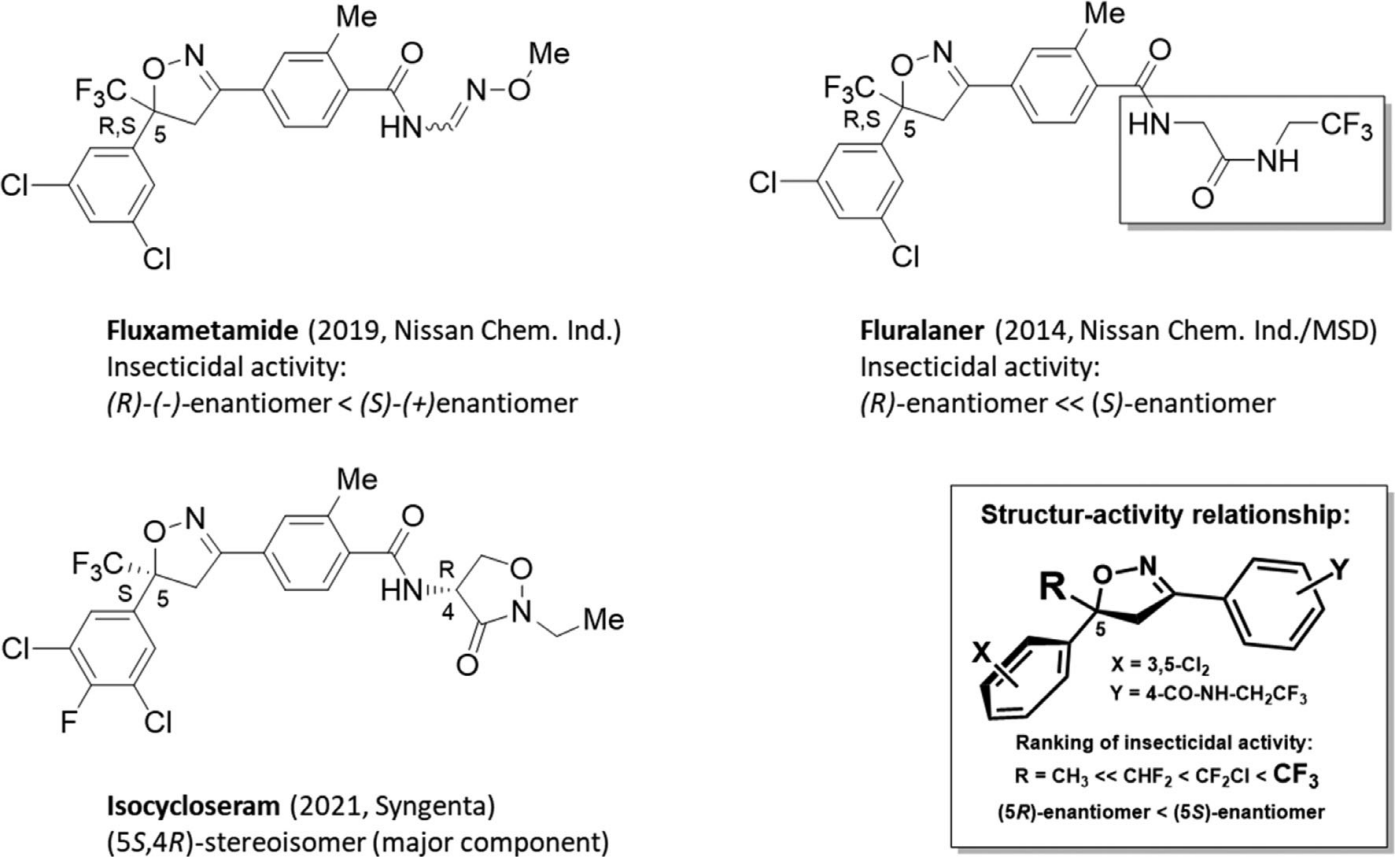
Structures of the racemic fluxametamide in comparison with the racemic fluralaner and isocycloseram as (5*S*,4*R*)‐stereoisomer (major component). Structure–activity relationship of isoxazoline insecticides depending on R in 5‐position of the 4,5‐dihydro‐isoxazole moiety.

The function of the GABA‐gated chloride channel is to regulate membrane electrical excitability, specifically, by causing neuronal inhibition. GABA is the most important inhibitory neurotransmitter in insects.^126^ To date, unfortunately, major insects evolved resistance to the first generation of non‐competitive antagonists (NCAs), the so‐called GABA‐gated chloride channel blockers (IRAC MoA main group 2) such as the cyclodiene (IRAC MoA sub‐group 2A) and phenylpyrazoles (fiproles) (IRAC MoA sub‐group 2B). Since 2019, however, the new IRAC MoA group 30 has been established, which contains three members and two of them are stereoisomeric isoxazolines such as fluxametamide (Section 6.3.1) and isocycloseram (Section 6.3.2) (Fig. 12).

These insecticides act on the nervous system of insects and allosterically inhibit the GABA‐activated chloride channel, leading to overexcitation and convulsions.

#### 6.3.1 Fluxametamide

The first member of isoxazolines within the new IRAC MoA group 30 is the racemic fluxametamide (2019, Gracia, Nissan Chemical Industries) (Fig. 12).^58^, ^127^, ^128^ It contains a *N*‐[(*E,Z*)‐(methoxyimino)methyl]‐2‐methylbenzamide moiety and demonstrates a broad activity against various lepidopteran, thysanopteran, and dipteran pest species, and shows acaricidal activity. The insecticidal efficacy *in vitro* of *rac*‐fluxametamide and both enantiomers has been studied on important agricultural insects such as diamondback moth (*Plutella xylostella*), cotton aphid (*Aphis gossypii*), and carmine spider mite (*Tetranychus cinnabarinus*). In this test *rac*‐fluxametamide and (*S*)‐(+)‐fluxametamide showed high activities toward these pests. The consistent order of activities against three insects was found to be *R*‐(−)‐fluxametamide < *rac*‐fluxametamide < (*S*)‐(+)‐fluxametamide (for chiral separation see Section 2.2, Table S1).^29^ Furthermore, acute contact toxicities against honey bees (*Apis mellifera*) increased in the order *R*‐(−)‐fluxametamide < (*S*)‐(+)‐fluxametamide < *rac*‐fluxametamide. Although *rac*‐fluxametamide had a 4.3‐fold higher acute contact toxicity in honeybees than (*S*)‐(+)‐fluxametamide, its toxicity is far lower than that of other GABA‐controlled chloride channel blockers such as fipronil. The lack of a low‐cost asymmetric manufacturing process for fluxametamide means that only the racemic mixture is preferably prepared, although the (*S*)‐(+)‐enantiomer is more active. This economic decision correlates well with the structurally related and commercial racemic isoxazoline ectoparasiticide fluralaner.

#### 6.3.2 Isocycloseram

Isocycloseram (2021, Plinazolin, Syngenta) (Fig. 12),^58^, ^129^ a mixture comprising 80–100% of the (5*S*,4*R*)‐stereoisomer and 20% to 0% the (5*R*,4*R*)‐, (5*R*,4*S*)‐ and (5*S*,4*S*)‐stereoisomers, is the second member of isoxazolines within the new IRAC MoA group 30 GABA‐gated chloride channel allosteric modulators.^130^ A matched molecular pair analysis (MMPA) for the aryl ring at the C‐5 stereogenic center of the 5‐trifluoromethylated 2‐isoxazoline moiety as well as multiparameter optimization and faster cycles of design synthesis test analysis (DSTA) was used to find isocycloseram. The cost‐efficient asymmetric manufacturing of the active isocycloseram (5*S*,4*R*)‐stereoisomer is described in Section 2.2 (see Table S2). The (*S*)‐configuration at this stereogenic center has a remarkable impact on biological activity for all commercialized isoxazolines.^129^ In the case of isoxazoline insecticides, the activity of the mixtures enriched with (*S*)‐enantiomer is higher than that of the respective racemic mixtures. As a broad‐spectrum insecticide and acaricide isocycloseram can be used for control of a range of pests on several crops such as lepidopteran, hemipteran, coleopteran, thysanopteran and dipteran pest species, with potential for both foliar and seed treatment applications. In the last year Syngenta Seedcare lauched Equento®, a new seed treatment based on isocycloseram. Equento® can be applied across multiple crops including cereals and canola and is intended to control a variety of soil pests, including wireworms and red‐legged earth mites. Recently it has been reported, that isocycloseram demonstrates effectiveness against ants, specifically leaf‐cutting ants, contributing to the development of efficient and environmentally safe ant baits.^131^

### 6.4 Selected chiral development candidate insecticides

Based on the structure of the dipolar compound class ‘mesoionics’ (inner salt and tautomers for the negative charge given), exemplified by the non‐chiral triflumezopyrim (2018, Pexalon, DuPont) (Fig. S3)^58^, ^132^ as a member of the *n*AChR competitive modulators from the IRAC MoA sub‐group 4E, fenmezoditiaz (ISO‐proposed, June 2021) (Fig. S3)^133^ has been developed as the first chiral compound.

As pure (*R*)‐enantiomer, fenmezoditiaz (Axalio) has excellent systemic properties and a broad insecticidal spectrum. It can be used in diverse application methods against piercing and sucking insects in a range of crops including cereals, root crops, vegetables, and ornamentals. Furthermore, it can control the rice hopper complex, and a lack of cross resistance in the neonicotinoid resistant brown planthopper (BPH) and small brown planthopper (SBPH) strains. Therefore, fenmezoditiaz can be an excellent tool of integrated pest management (IPM) and IRM, especially for the rice hopper complex.

## 7 ACARICIDES

Acaricides are mandatory for the efficient control of phytophagous mites. According to their significance to the global acaricide market, spider mites in genera such as two‐spotted spider mite (*Tetranychus urticae*), citrus red mite (*Panonychus citri*) and European red mite (*Panonychus ulmi*) are the most important mite species causing severe damage to a broad range of crops including fruits, vegetables, and tea.

### 7.1 Calcium‐activated potassium channel (K_Ca_2) modulators

The new IRAC MoA main group 33 contains a.i.s acting on nerve and muscle targets with negative modulation of calcium‐activated potassium channel (K_Ca_2) in insects causing hyperexcitation and convulsions. The K_Ca_2 channels are activated by an increase of the intracellular calcium concentration and are involved in the regulation of action potentials. By using electrophysiological techniques (patch‐clamp) it has been found, that the new chiral acaricide acynonapyr (see Section 7.1.1) modulates the activity of K_Ca_2 channels in the two‐spotted spider mite (*T. urticae*).^134^

#### 7.1.1 Acynonapyr

**Figure 13:**
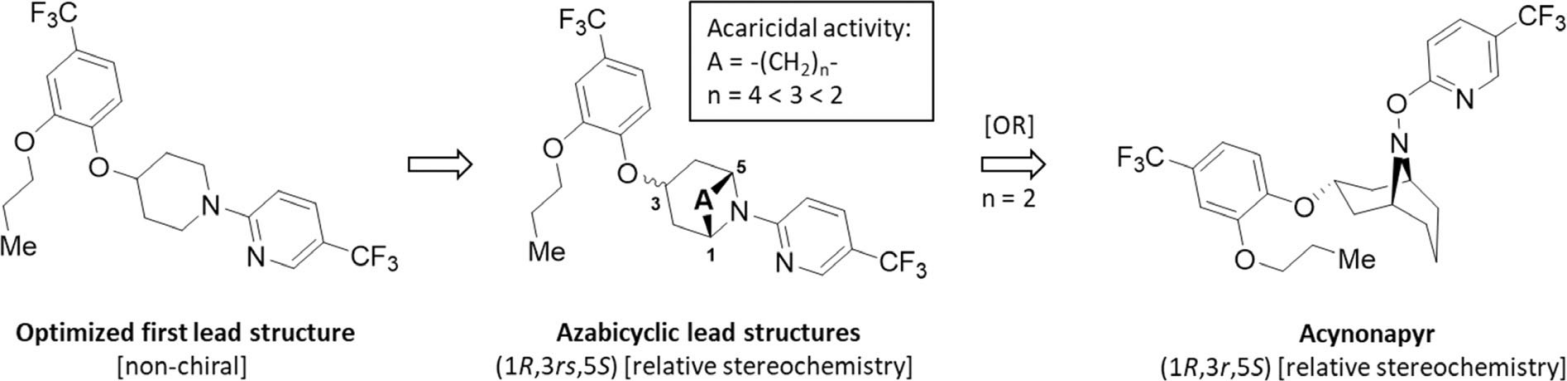
Further optimization of the first lead structure and resulting azabicyclic lead structures leading to the acaricide acynonapyr; OR, oxidative rearrangement, proposed by Hamamoto *et al*.^135^

Starting with acaricidal active, non‐chiral 1‐[(hetero)aryl]piperidine derivatives represented by the optimized first lead structure, chiral 3‐*endo*/3‐*exo*‐azabicyclic lead structures were designed having a relative stereochemistry (Fig. 13).^58^, ^135^

The relative configuration is the experimentally determined relationship between two enantiomers, even if the absolute configuration is not known. By optimization of the [–(CH_2_)_*n*_–] bridge element A in the tropane ring it was found, that the ethylene group (*n* = 2) leads to the best acaricidal efficacy. Surprisingly, oxidation of this tropane by *meta*‐chloroperbenzoic acid resulted not in the expected amine *N*‐oxide, but in the *N*‐pyridyl‐oxyamine acynonapyr. Possibly, the *in situ* formed amine *N*‐oxide rearranged to 3‐*endo*‐(1*R*,3*R*,5*S*)‐stereoisomers acynonapyr via an oxidative rearrangement (OR) similar to the Meisenheimer rearrangement. Acynonapyr (Danyote, 2020, Nippon Soda) (Fig. 13)^135^ blocks *T. urticae* calcium‐activated potassium channels (TurK_Ca_2) in a concentration‐dependent manner. Finally, a comparison of its acaricidal activity against *T. urticae* with inhibitory activity against TurK_Ca_2 revealed that these channels are the primary toxicological targets.^134^ The acaricide acynonapyr, is targeted at use on fruit and vegetables and tea and is reported to have no adverse effects on beneficial insects. In 2020, the product Daniote Flowable® has been launched in Japan for the selective control of spider mites (*T. urticae*) and the European red mite (*Panonychus ulmi*) on fruit and vegetable crops, including citrus, apple, pear, strawberry, watermelon, and eggplant.

## 8 NEMATICIDES

Today, agriculture around the world is challenged by plant parasitic nematode (PPN) infections. PPNs are destructive pathogens that can cause significant damages and yield losses of up to 12% globally per year, equating to an estimated loss of $150 billion every year for farmers and are a threat to food security. For decades, synthetic nematicides have been important in PPN control. In the meantime, however, concerns about environmental toxicity and human safety have led to the fact that the numerous nematicides are no longer permitted.^136^ The lack of available nematicides for PPN control is enormous. Of the top 20 nematicides used in the 20th century, only four are currently approved for use in the European Union and only three in the United States without restriction.^136^ While these withdrawals are justified, they leave farmers with limited opportunities for satisfactory nematode control. Therefore, there is an urgent need for new nematicides with improved selectivity.^137^

### 8.1 Cyclobutrifluram

The nematicide cyclobutrifluram (2022, Victrato, Syngenta Seedcare)^138^ has been classified together with fluopyram^86^ in the nematicide MoA classification scheme in group N‐3 as mitochondrial complex II electron transport inhibitors – succinate‐coenzyme Q reductase inhibitors (Table 4). Victrato contains the Tymirium® technology (see Section 5.1.3),^37^ which provides long‐lasting protection against a broad spectrum of parasitic nematodes and fungal diseases across major crops, including soybeans, corn, cereal, cotton, and rice. Cyclobutrifluram targets root‐knot nematode (RKN), cabbage cyst nematodes and maize short body nematodes on crops such as cucumber, tomato, corn, and sugar beet. It can be used for soil treatment or seed treatment, long‐term control of nematodes and diseases in major crops and various environments. By using the nematode *Caenorhabditis elegans* as a model organism, it has been shown that cyclobutrifluram strongly impacts the survival and fertility rates of the nematode by decreasing the number of germ cells. A genetic approach demonstrated, that cyclobutrifluram functions by inhibiting the mitochondrial SDH complex. Transcriptomic analysis revealed a strong response to its exposure.^139^

## 9 SUMMARY AND PROSPECTS

The continuing significance of chiral agrochemicals from the development pipeline has demonstrated that these crop protection products can have significant agricultural impact. According to the subdivision for agrochemicals, fungicides introduced in the past 6 years contain the largest number of chiral compounds, followed by insecticides/acaricides/nematicides, and herbicides. Due to the continued importance of SDH inhibitor fungicides, products with an improved fungicidal efficacy profile are becoming increasingly important. The agrochemical industry is still interested in searching for biologically active and easily accessible chiral natural products. It has been shown that selective, structural modifications can lead to new and innovative chiral agrochemicals through a semi‐synthetic approach. This innovative approach was again proven with the natural products pyripyropene A and UK‐2A. While the insecticide afidopyropen (Meiji/BASF) was developed from pyripyropene A, the two pro‐fungicides fenpicoxamid and florylpicoxamid, which are suitable for disease control for the grain market, could be obtained from the natural product UK‐2A. It has been shown once again that most chiral agrochemicals will continue to be produced as racemates in the future due to cost‐efficient asymmetric technologies. This trend is evident in herbicides (e.g., tiafenacil), fungicides (e.g., mefentrifluconazole, fluindapyr, fluoxapiprolin) and insecticides (e.g., fluxametamide, dimpropyridaz). In the future, it could be important to develop industrial manufacturing processes for commercial chiral stereoisomers that are currently used as a racemic mixture. Furthermore, the improvement of biocatalytic pathways, which include both enzymatic dissolutions and asymmetric (bio)catalysis, will be of crucial importance for the agrochemical industry.

## Supporting information


**Figure S1.** Chiral development candidate herbicides: protoporphyrinogen IX oxidase (PPO) inhibitor flufenoximacil, auxin inhibitor fluchchloraminopyr and its proherbicide fluchchloraminopyr‐tefuryl, 4‐hydroxy‐phenylpyrovate dioxygenase (4‐HPPD) inhibitor flusulfinam as (*R*)‐enantiomer (major component), fatty acid thioesterase (FAT) inhibitor cinflubrolin as mixture of the (1*S*,2*R*,4*R*)‐ and (1*R*,2*S*,4*S*)‐stereoisomers, and the plant tubulin polymerization inhibitor icafolin and its proherbicide icafolin‐methyl as mixture of the (2*R*,4*R*)‐ and (2*S*,4*S*)‐stereoisomers. (ISO‐proposed common names.)
**Figure S2.** Chiral development candidate fungicides: fungicidal quinone inside inhibitors (QiIs) metarylpicoxamid as (1*S*,2*S*,4*S*)‐stereoisomer, and the racemic sterol biosynthesis inhibitor (SBI) fluoxytioconazole. (ISO‐proposed common names.)
**Figure S3.** Chiral development candidate insecticdes: insecticidal mesoinonic triflumezopyrim as nAChR competitive modulator from the IRAC MoA sub‐group 4E has been the starting point for development of fenmezoditiaz as (*R*)‐enantiomer. (ISO‐proposed common name.)

**Table S1.** Chiral separation, preparation and detection methods of enantiomers.
**Table S2.** Catalytic asymmetric syntheses of agrochemical intermediates and final products launched between 2018 and 2023.
**Table S3.** The provision of starting materials for chiral fungicides and insecticides is based on fermentation of natural products.

## Data Availability

The data that support the findings of this study are available from the corresponding author upon reasonable request.

## References

[1] Gerwick BC and Sparks TC , Natural products for pest control: an analysis of their role, value and future. Pest Manag Sci 70:1169–1185 (2014). 24478254 10.1002/ps.3744

[2] Musarurwa H and Tavengwa N , Green aspects during synthesis, application and chromatographic analysis of chiral pesticides. Trends Environ Anal Chem 27:e00093 (2020).

[3] Federsel H‐J , Asymmetry on large scale: the roadmap to stereoselective processes. Nature Rev 4:685–697 (2005). 10.1038/nrd1798 16041317

[4] Cossy JR , Introduction: The Importance of Chirality in Drugs and Agrochemicals, in Comprehensive Chirality, Vol. 1, 1st edn, ed. by Yamamoto H and Carreira EM . Elsevier, Amsterdam, Netherlands, pp. 1–7 (2012).

[5] McVickerand RU and O'Boyle NM , Chirality of new drug approvals (2013−2022): trends and perspectives. J Med Chem 67:2305–2320 (2024). 38344815 10.1021/acs.jmedchem.3c02239 PMC10895675

[6] Patel BK and Hutt AJ , in Stereoselectivity in drug action and disposition: on overview in chirality in drug design and development, ed. by Reddy IK and Mehvar R . Marcel Dekker New York, pp. 139–190 (2004).

[7] Tombo GMR and Bellus D , Chiralität und Pflanzenschutz. Angew Chem 103:1219–1241 (1991).

[8] Spindler F and Blaser H‐U , The development of enantioselective catalytic processes for the manufacture of chiral intermediates for agrochemicals. Enantiomer 4:557–568 (1999).

[9] Williams A , Opportunities for chiral agrochemicals. Pestic Sci 46:3–9 (1996).

[10] Crosby J , Manufacture of optically active materials: an agrochemical perspective. Pestic Sci 46:11–31 (1996).

[11] Kurihara N and Miyamoto J eds, Chirality in agrochemicals. John Wliley & Sons, New York (2003).

[12] Wendeborn S , Godineau E , Mondiere R , Smejkal T and Smits H , Chirality in agrochemicals, in Comprehensive Chirality, Vol. 1, 1st edn, ed. by Yamamoto H and Carreira EM . Elsevier, Amsterdam, Netherlands, pp. 120–166 (2012).

[13] Garrison A , Gan J and Liu W , ACS symposium series, in Chiral Pesticides: Stereoselectivity and its Consequences, Vol. 1085. American Chemical Society, Washington, DC, pp. 1–7 (2011).

[14] Jeschke P , Current status of chirality in agrochemicals. Pest Manag Sci 74:2389–2404 (2018). 29704299 10.1002/ps.5052

[15] Jeschke P and Agrochemicals C , in Agricultural Biocatalysis: Biological and Chemical Applications, Vol. 10, ed. by Jeschke P and Starikov EB . Jenny Stanford Publishing Pte. Ltd., Singapore, pp. 261–309 (2023).

[16] Ventura‐Hernández KI , Delgado‐Alvarado E , Janardan Pawar TJ and Olivares‐Romero JL , Chirality in insecticide design and efficacy. J Agric Food Chem 72:20722–20737 (2024). 39255417 10.1021/acs.jafc.4c05363

[17] Ye J , Zhao M , Niu L and Liu W , Enantioselective environmental toxicology of chiral pesticides. Chem Res Toxicol 28:325–338 (2015). 25643169 10.1021/tx500481n

[18] Ma Y and Liu GW , Chiral pesticides and environmental safety, in Chiral Pesticides: Stereoselectivity and its Consequence, ed. by Garrison AW , Gan J and Liu W . ACS, Washington, USA, pp. 97–106 (2016).

[19] Chen D , Hao G and Song B , Finding the missing property concepts in pesticide‐likeness. J Agric Food Chem 70:10090–10099 (2022). 35971945 10.1021/acs.jafc.2c02757

[20] Kesh M and Goel S , Target‐based screening for lead discovery, in CADD and Informatics in Drug Discovery. Interdisciplinary Biotechnological Advances, ed. by Rudrapal M and Khan J . Springer, Singapore, pp. 141–173 (2023).

[21] Lorenz H and Seidel‐Morgenstern A , Processes to separate enantiomers. Angew Chem Int Ed 53:1218–1250 (2014). 10.1002/anie.201302823 24442686

[22] Peluso P and Chankvetadze B , Recognition in the domain of molecular chirality: from noncovalent interactions to separation of enantiomers. Chem Rev 122:13235–13400 (2022). 35917234 10.1021/acs.chemrev.1c00846

[23] Jeschke P , New active ingredients for sustainable modern chemical crop protection in agriculture. ChemSusChem e202401042 (2024). 10.1002/cssc.202401042 PMC11739819 39373399

[24] Qian H‐L , Xu S‐T and Yan X‐P , Recent advances in separation and analysis of chiral compounds. Anal Chem 95:304–318 (2023). 36625130 10.1021/acs.analchem.2c04371

[25] Huang XY , Pei D , Liu JF and Di DL , A review on chiral separation by counter‐current chromatography: development, applications and future outlook. J Chromatogr A 1531:1–12 (2018). 29173957 10.1016/j.chroma.2017.10.073

[26] Matsunaga T , Hiraguri N , Takahashi T , Inui T and Tanimoto M , Method for Producing (R)‐1,1,3‐Trimethyl‐4‐Aminoindane. PCT Int. Appl. WO 2015/118793. Sumitomo Chem. Comp. Ltd., (2015).

[27] Tong Z , Dong X , Meng DD , Yi XT , Sun MN , Chu Y *et al*., Enantioselective degradation and bioactivity mechanism of a new chiral fungicide fluindapyr in paddy ecosystems. J Agric Food Chem 71:1426–1433 (2023). 36630283 10.1021/acs.jafc.2c07924

[28] Li L , Sun X , Zhao X , Xiong Y , Gao B , Zhang J *et al*., Absolute configuration, enantioselective bioactivity, and degradation of the novel chiral triazole fungicide mefentrifluconazole. J Agric Food Chem 69:4960–4967 (2021). 33877830 10.1021/acs.jafc.0c07947

[29] Li R , Pan X , Wang Q , Tao Y , Chen Z , Jiang D *et al*., Development of S‐fluxametamide for bioactivity improvement and risk reduction: systemic evaluation of the novel insecticide fluxametamide at the enantiomeric level. Environ Sci Technol 53:13657–13665 (2019). 31684725 10.1021/acs.est.9b03697

[30] Tian X , Chen H , Liu H and Chen J , Recent advances in lactic acid production by lactic acid bacteria. Appl Biochem Biotechnol 193:4151–4171 (2021). 34519919 10.1007/s12010-021-03672-z

[31] Jeanmart S , Edmunds AJF , Lamberth C , Pouliot M and Morris JA , Synthetic approaches to the 2015‐2018 new agrochemicals. Bioorg Med Chem 39:116162 (2021). 33895705 10.1016/j.bmc.2021.116162

[32] Yang X , Jiang S , Jin Z and Tingting L , Application of asymmetric catalysis in chiral pesticide active molecule synthesis. J Agric Food Chem 72:17153–17165 (2024). 39051451 10.1021/acs.jafc.4c02343

[33] Tamatam R and Shin D , Asymmetric synthesis of US‐FDA approved drugs over five years (2016–2020): a recapitulation of chirality. Pharmaceuticals 16:339 (2023). 36986439 10.3390/ph16030339 PMC10052577

[34] Ogawa N , Toyoshima S , Sekikawa S , Ishijima M , Katagiri K , Uematsu C *et al*., Synthesis and herbicidal activity of optically active cinmethylin, its enantiomer, and C3‐substituted cinmethylin analogs. J Pestic Sci 48:11–16 (2023). 36874636 10.1584/jpestics.D22-051 PMC9978247

[35] Chen Y , Pyrrolidinones and a process to prepare them WO2018/175226, 2018 (FMC Corporation).

[36] Jeanmart S , Edmunds AJF , Lamberth C , Pouliot M and Morris JA , Synthetic approaches to the 2019–2020 new agrochemicals. Synthesis 56:357–367 (2024).

[37] Loiseleur O , TYMIRIUM technology: the discovery of cyclobutrifluram, in 15th IUPAC International Congress of Crop Protection Chemistry. New Delhi, India, pp. 14–17 (2023).

[38] Hughes DL , Highlights of the recent patent literature: focus on asymmetric organocatalysis. Org Process Res Dev 26:2224–2239 (2022).

[39] Goetz R , Rack M , McLaughlin MJ , Shinde H , Karalkar R , Borate K *et al*., Process for Preparation of Optically Enriched Isoxazolines WO 2020/094434, 2020 (BASF SE).

[40] Hughes GJ and Lewis JC , Introduction: biocatalysis in industry. Chem Rev 118:1–3 (2018). 29316793 10.1021/acs.chemrev.7b00741

[41] Gröger H and Dranz K , Methods for enantioselective biocatalytic production of L‐amino acids on an industrial scale, in Asymmetric Catalysis on Industrial Scale: Challenges, Approaches and Solutions, ed. by Blaser HU and Schmidt E . Germany, Wiley‐VCH, Weinheim, pp. 131–147 (2004).

[42] Marić I , Guo Y , Fürst MJLJ , Van Aelst K , Van den Bosch S , De Simone M *et al*., A one‐pot, whole‐cell biocatalysis approach for vanillin production using lignin oil. Adv Synth Catal 365:3987–3995 (2023).

[43] Ueki M , Abe K , Hanafi M , Shibata K , Tanaka T and Taniguchi M , UK‐2A, B, C, and D, novel antifungal antibiotics from *Streptomyces* sp. 517‐02. I. Fermentation, isolation, and biological properties. J Antibiotics 49:639–643 (1996). 10.7164/antibiotics.49.639 8784423

[44] Horikoshi R , Goto K , Mitomi M , Oyama K , Hirose T , Sunazuka T *et al*., Afidopyropen, a novel insecticide originating from microbial secondary extracts. Sci Rep 12:2827 (2022). 10.1038/s41598-022-06729-z. 35181691 PMC8857236

[45] Kwon OE , Rho M‐C , Song HY , Lee SW , Chung MY , Lee JH *et al*., Phenylpyropene A and B, new inhibitors of acyl‐CoA: cholesterol acyltransferase produced by *Penicillium griseofulvum* F1959. J Antibiot 55:1004–1008 (2002). 10.7164/antibiotics.55.1004 12546421

[46] Hu J , Okawa H , Yamamoto K , Oyama K , Mitomi M and Anzai H , Characterization of two cytochrome P450 monooxygenase genes of the pyripyropene biosynthetic gene cluster from *Penicillium coprobium* . J Antibiot 64:221–227 (2011). 10.1038/ja.2010.162 21224862

[47] Wang F , Li X , Jiang S , Han J , Wu J , Yan M *et al*., Enantioselective behaviors of chiral pesticides and enantiomeric signatures in foods and the environment. J Agric Food Chem 71:12372–12389 (2023). 37565661 10.1021/acs.jafc.3c02564

[48] Gehen S , Corvaro M , Jones J , Ma M and Yang Q , Challenges and opportunities in the global regulation of crop protection products. Org Process Res Dev 23:2225–2233 (2019).

[49] United States Environmental Protection Agency EPA ). https://www.epa.gov/pesticide-science-and-assessing-pesticide-risks/interim-policy-evaluation-stereoisomeric-pesticides. (accessed 02 November 2024).

[50] Lewis DL , Garrison AW , Wommack KE , Whittemore A , Steudler P and Melillo J , Influence of environmental changes on degradation of chiral pollutants in soils. Nature 401:898–901 (1999). 10553905 10.1038/44801

[51] García‐Cansino L , María Luisa Marina ML, and García MA, chiral analysis of pesticides and emerging contaminants by capillary electrophoresis ‐ application to toxicity evaluation. Toxics 12:185 (2024). 38535919 10.3390/toxics12030185 PMC10974875

[52] Meng Z , Cui J , Lib R , Sun W , Bao X , Wang J *et al*., Systematic evaluation of chiral pesticides at the enantiomeric level: a new strategy for the development of highly effective and less harmful pesticides. Sci Total Environ 846:157294 (2022). 35839878 10.1016/j.scitotenv.2022.157294

[53] Maia AS , Ribeiro AR , Castro PML and Tiritan ME , Chiral analysis of pesticides and drugs of environmental concern: biodegradation and enantiomeric fraction. Symmetry 9:196 (2017).

[54] Beffa R , Menne H and Köcher H , Herbicide resistance action committee (HRAC): herbicide classification, resistance evolution, survey, and resistance migation activities, in Modern Crop Protection Compounds, Vol. 1. Herbicides, 3rd edn, ed. by Jeschke P , Witschel M , Krämer W and Schirmer U . Wiley‐VCH, Weinheim, Germany, pp. 5–32 (2019).

[55] Hermann D and Stenzel K , FRAC mode‐of‐action classification and resistance risk of fungicides, in Modern Crop Protection Compounds, Vol. 2. Herbicides, 3rd edn, ed. by Jeschke P , Witschel M , Krämer W and Schirmer U . Wiley‐VCH, Weinheim, Germany, pp. 589–608 (2019).

[56] Nauen R , Slater R , Sparks TC , Elbert A and McCaffery A , IRAC: insecticide resistance and mode‐ of‐action classification of insecticides, in Modern Crop Protection Compounds, Vol. 3, Insecticides, 3rd edn, ed. by Jeschke P , Witschel M , Krämer W and Schirmer U . Wiley‐VCH, Weinheim, Germany, pp. 995–1012 (2019).

[57] Zagar C , Liebl R , Theodoridis G and Witschel M , Protoporphyrinogen IX oxidase inhibitors, in Modern Crop Protection Compounds, Vol. 1, Herbicides, 3rd edn, ed. by Jeschke P , Witschel M , Krämer W and Schirmer U . Wiley‐VCH, Weinheim, Germany, pp. 173–211 (2019).

[58] Jeschke P , Recent developments in fluorine‐containing pesticides. Pest Manag Sci 80:3065–3087 (2024). 38073050 10.1002/ps.7921

[59] Alnafta N , Beffa R , Bojack G , Bollenbach‐Wahl B , Brant NZ , Dörnbrack C *et al*., Designing new protoporphyrinogen oxidase‐inhibitors carrying potential side chain isosteres to enhance crop safety and spectrum of activity. J Agric Food Chem 71:18270–18284 (2023). 37269295 10.1021/acs.jafc.3c01420

[60] Mattison RL , Beffa R , Bojack G , Bollenbach‐Wahl B , Dörnbrack C , Dorn N *et al*., Design, synthesis and screening of herbicidal activity for new phenyl pyrazole‐based protoporphyrinogen oxidase‐inhibitors (PPO) overcoming resistance issues. Pest Manag Sci 79:2264–2280 (2023). 36815643 10.1002/ps.7425

[61] Park J , Ahn YO , Nam JW , Hong MK , Song N , Kim T *et al*., Biochemical and physiological mode of action of tiafenacil, a new protoporphyrinogen IX oxidase‐inhibiting herbicide. Pestic Biochem Physiol 152:38–44 (2018). 30497709 10.1016/j.pestbp.2018.08.010

[62] Jing F , Cantu D , Tvaruzkova J , Chipman J , Nikolau B , Yandeau‐Nelson M *et al*., Phylogenetic and experimental characterization of an acyl‐ACP thioesterase family reveals significant diversity in enzymatic specificity and activity. BMC Biochem 12:44 (2011). 21831316 10.1186/1471-2091-12-44 PMC3176148

[63] Peng Z , Zhang H , Tian H , Shan L , Zhang Z , Ding H *et al*., The phylogeny and functional C characterization of peanut acyl‐ACP thioesterases. J Plant Growth Regul 39:1381–1392 (2020).

[64] Campe R , Hollenbach E , Kämmerer L , Hendriks J , Höffken HW , Kraus H *et al*., A new herbicidal site of action: cinmethylin binds to acyl‐ACP thioesterase and inhibits plant fatty acid biosynthesis. Pestic Biochem Physiol 148:116–125 (2018). 29891362 10.1016/j.pestbp.2018.04.006

[65] He B , Hu Y , Wang W , Yan W and Ye Y , The Progress towards novel herbicide modes of action and targeted herbicide development. Agronomy 12:2792 (2022). 10.3390/agronomy12112792.

[66] Jung J , Schmölzer K , Schachtschabel D , Speitling M and Nidetzky B , Selective β‐mono‐glycosylation of a C15‐hydroxylated metabolite of the agricultural herbicide cinmethylin using Leloir glycosyltransferases. J Agric Food Chem 69:5491–5499 (2012). 10.1021/acs.jafc.1c01321 PMC8278484 33973475

[67] Comont D , Crook L , Hull R , Sievernich B , Kevisc S and Nevea P , The role of interspecific variability and herbicide pre‐adaptation in the cinmethylin response of *Alopecurus myosuroides* . Pest Manag Sci 80:3172–3181 (2024). 38345468 10.1002/ps.8021

[68] Dayan FE , Current status and future prospects in herbicide discovery. Plan Theory 8:341 (2019). 10.3390/plants8090341 PMC6783942 31514265

[69] Zrenner R , Stitt M , Sonnewald U and Boldt R , Pyrimidine and purine biosynthesis and degradation in plants. Annu Rev Plant Biol 57:805–836 (2006). 16669783 10.1146/annurev.arplant.57.032905.105421

[70] Ullrich A , Knecht W , Piskur J and Löffler M , Plant dihydroorotate dehydrogenase differs significantly in substrate specificity and inhibition from the animal enzymes. FEBS Lett 529:346–350 (2002). 12372626 10.1016/s0014-5793(02)03425-7

[71] Selby TP , Satterfield AD , Puri A , Stevenson TM , Travis DA , Campbell MJ *et al*., Bioisosteric tactics in the discovery of tetflupyrolimet: a new mode‐of‐action herbicide. J Agric Food Chem 71:18197–18204 (2023). 37285594 10.1021/acs.jafc.3c01634

[72] Kanga IH , Emptagea RP , Kima SI and Gutteridgea S , A novel mechanism of herbicide action through disruption of pyrimidine biosynthesis. Proceedings of the National Academy of Sciences 120:e2313197120 (2023). 10.1073/pnas.2313197120 PMC10691210 37988466

[73] Wong N , China's three new novel pesticides to be registered first time, in AgNews. Chongqing Stanley Info‐tech Co., Ltd. (2024) https://news.agropages.com/News/NewsDetail---50163.htm. (accessed 05 November 2024).

[74] Jeschke P , Propesticides and their use as agrochemicals. Pest Manag Sci 72:210–225 (2016). 26449612 10.1002/ps.4170

[75] Lian L , Peng X , Hua R , Zhang J and Cui Q , R‐Type Pyridyloxycarbonylic Acid, Salt and Ester Derivative Thereof, and Preparation Method Therof, and Herbicidal Composition and Application Thereof. PCT Int. Appl. WO 2020/135235. Dao Kingagroot Chemical Compound Co., (2020).

[76] Liu S , Li X , Zhu J , Liang L , Zhang H , Liao Y *et al*., Novel herbicide flusulfinam: absolute configuration, enantioseparation, enantioselective bioactivity, toxicity and degradation in paddy soils. Pest Manag Sci 80:5244–5255 (2024). 10.1002/ps.8251. 39031670

[77] Liu S , Li X , Zhang H , Qin S , Liang L , Liao Y *et al*., Comprehensive study of chiral herbicide flusulfinam uptake, translocation, degradation, and subcellular distribution in rice (*Oryza sativa* L.). Pestic Biochem Physiol 204:106018 (2024). 39277354 10.1016/j.pestbp.2024.106018

[78] ISO approved two new pesticide common names: Cinflubrolin and Cybenzoxasulfyl AgNews (2024); https://news.agropages.com/News/NewsDetail---50924.htm. (accessed 05 November 2024).

[79] Haaf K‐H , Peters O , Laber B , Lange G , Gatzweiler E , Geibel S *et al*., The novel herbicide icafolin‐methyl is a plant‐specific inhibitor of tubulin polymerization. Pest Manag Sci (2024). 10.1002/ps.8415. 39297346

[80] Rheinheimer J , Succinate dehydrogenase inhibitors, in Modern Crop Protection Compounds, Vol. 2, Fungicides, 3rd edn, ed. by Jeschke P , Witschel M , Krämer W and Schirmer U . Wiley‐VCH, Weinheim, Germany, pp. 681–694 (2019).

[81] Sierotzki H and Scalliet G , A review of current knowledge of resistance aspects for the next‐generation succinate dehydrogenase inhibitor fungicides. Phytopathology 103:880–887 (2013). 23593940 10.1094/PHYTO-01-13-0009-RVW

[82] Kiguchi S , Inoue T , Matsuzaki Y , Iwahashi F and Sakaguchi H , Discovery and biological profile of inpyrfluxam: a new broad‐spectrum succinate dehydrogenase inhibitor fungicide, in Recent Highlights in the Discovery and Optimization of Crop Protection Products, ed. by Maienfisch P and Mangelinckx S . Amsterdam, Academic Press, pp. 381–389 (2021).

[83] Grichar WJ and Meador CB , Using inpyrfluxam to control peanut (*Arachis hypogaea* L.) foliar and soil‐borne diseases. Int j exp agric 45:35–46 (2023).

[84] Adachi T , Suzuki Y and Fujisawa T , Photodegradation of an anilide fungicide inpyrfluxam in water and nitrate aqueous solution. J Agric Food Chem 69:12966–12973 (2021). 34699205 10.1021/acs.jafc.1c03813

[85] Guo P , An X , Chen W , Pan X , Li R , Xu R *et al*., Separation and determination of fluindapyr enantiomers in cucumber and tomato and by supercritical fluid chromatography tandem mass spectrometry. Food Chem 395:133571 (2022). 35802974 10.1016/j.foodchem.2022.133571

[86] Lümmen P and Fürsch H , Fluopyram a novel nematicide for the control of root‐knot‐nematodes, in Modern Crop Protection Compounds, Vol. 3, Insecticides, 3rd edn, ed. by Jeschke P , Witschel M , Krämer W and Schirmer U . VCH‐Wiley, Weinheim, Germany, pp. 1630–1643 (2019).

[87] Neft N and Farley TM , Conditions influencing antimycin production by a *Streptomyces* species grown in chemically defined medium. Antimicrob Agents Chemother 1:274–276 (1972). 4558141 10.1128/aac.1.3.274 PMC444205

[88] Huang LS , Cobessi D , Tung EY and Berry EA , Binding of the respiratory chain inhibitor antimycin to the mitochondrial bc1 complex: a new crystal structure reveals an altered intramolecular hydrogen‐bonding pattern. J Mol Biol 351:573–597 (2005). 16024040 10.1016/j.jmb.2005.05.053 PMC1482829

[89] Dong Y , Li B , Yin M‐X , Liu Z , Niu Y , Wu Q‐Y *et al*., The interaction mechanism of picolinamide fungicide targeting on the cytochrome bc1 complex and its structural modification. J Agric Food Chem 72:3755–3762 (2024). 38346446 10.1021/acs.jafc.3c05982

[90] Owen WJ , Yao C , Myung K , Kemmitt G , Leader A , Meyer KG *et al*., Biological characterization of fenpicoxamid, a new fungicide with utility in cereals and other crops. Pest Manag Sci 73:2005–2016 (2017). 28471527 10.1002/ps.4588 PMC5599960

[91] Shibata K , Hanafi M , Fujii J , Sakenaka O , Iinuma A , Ueki M *et al*., UK‐2A, B, C and D, novel antifungal antibiotics from *Streptomyces* sp. 517‐02. III. Absolute configuration of an antifungal antibiotic, UK‐2A, and consideration of its conformation *J* . Antibiot 51:1113–1116 (1998). 10.7164/antibiotics.51.1113 10048572

[92] Shimano M , Kamei N , Shibata T , Inoguchi K , Itoh N , Ikari T *et al*., Total synthesis of the antifungal dilactones UK‐2A and UK‐3A: the determination of their relative and absolute configurations, analog synthesis and antifungal activities. Tetrahedron 54:12745–12774 (1998).

[93] Owen WJ , Meyer KG , Meyer ST , Li F , Slanec TJ , Wang NX *et al*., Synthesis and biological activity of analogs of the antifungal antibiotic UK‐2A. II. Impact of modifications to the macrocycle benzyl position. Pest Manag Sci 75:1831–1846 (2019). 30636031 10.1002/ps.5329

[94] Owen WJ , Meyer KG , Slanec TJ , Meyer ST , Wang NX , Fitzpatrick GM *et al*., Synthesis and biological activity of analogs of the antifungal antibiotic UK‐2A. III. Impact of modifications to the macrocycle isobutyryl ester position. Pest Manag Sci 76:277–286 (2020). 31207132 10.1002/ps.5511

[95] Machida K , Takimoto H , Miyoshi H and Taniguchi M , UK‐2A, B, C and D, novel antifungal antibiotics from *Streptomyces* sp. 517.02. V. Inhibition mechanism of bovine heart mitochondrial cytochrome bc1 by the novel antibiotic UK‐2A. J Antibiot 52:748–753 (1999). 10.7164/antibiotics.52.748 10580388

[96] Wender PA , Verma VA , Paxton PJ and Pillow PH , Function‐oriented synthesis, step economy, and drug design. Acc Chem Res 41:40–49 (2008). 18159936 10.1021/ar700155p

[97] Meyer KG , Bravo‐Altamirano K , Herrick J , Loy BA , Yao C , Nugent B *et al*., Discovery of florylpicoxamid, a mimic of a macrocyclic natural product. Bioorg Med Chem 50:116455 (2021). 34757295 10.1016/j.bmc.2021.116455

[98] Babij NR , Choy N , Cismesia MA , Couling DJ , Hough NM , Johnson PL *et al*., Design and synthesis of florylpicoxamid, a fungicide derived from renewable raw materials. Green Chem 22:6047–6054 (2020).

[99] Meyer KG , Yao C , Lu Y , Bravo‐Altamirano K , Buchan Z , Daeuble JF *et al*., The discovery of florylpicoxamid, a new picolinamide for disease control, in Recent Highlights in the Discovery and Optimization of Crop Protection Products, ed. by Maienfisch P and Mangelinckx S . Amsterdam, Academic Press, pp. 433–442 (2021).

[100] Jaswal A , Singh PP and Mondal T , Furfural – a versatile, biomass‐derived platform chemical for the production of renewable chemicals. Green Chem 24:510–551 (2022).

[101] Yao C , Meyer KG , Gallup C , Bowling AJ , Hufnagl A , Myung K *et al*., Florylpicoxamid, a new picolinamide fungicide with broad spectrum activity. Pest Manag Sci 77:4483–4496 (2021). 34010509 10.1002/ps.6483

[102] Cohen Y , Rubin AE and Galperin M , Oxathiapiprolin‐based fungicides provide enhanced control of tomato late blight induced by mefenoxam‐insensitive *Phytophthora infestans* . PLoS One 13:e0204523 (2018). 30260986 10.1371/journal.pone.0204523 PMC6160094

[103] Saheki Y and DeCamilli P , Endoplasmic reticulum‐plasmamembrane contact sites. Annu Rev Biochem 86:659–684 (2017). 28301744 10.1146/annurev-biochem-061516-044932

[104] Pasteris RJ , Hoffman LE , Sweigard JA , Andreassi JL , Ngugi HK , Perotin B *et al*., Oxysterol‐binding protein inhibitors: oxathiapiprolin – a new omycete fungicide that targets an oxysterol‐binding protein, in Modern Crop Protection Compounds, Vol. 2, Fungicides, 3rd edn, ed. by Jeschke P , Witschel M , Krämer W and Schirmer U . VCH‐Wiley, Weinheim, Germany, pp. 979–987 (2019).

[105] Liang X , Su W , Chang AK , Zhuang C , Pei Y , Ai J *et al*., Toxicokinetics of two oxathiapiprolin enantiomers in rats and their stereoselective interaction with oxysterol binding protein. J Agric Food Chem 70:12180–12188 (2022). 36121774 10.1021/acs.jafc.2c02882

[106] Bryant R and Bite M eds, AG Chem New Compounds Review, Vol. 39. Agranova, UK (2021).

[107] Li C , Liu X , Liu Z , Hu S , Xue Z , Fu Y *et al*., Resistance risk and novel resistance‐related point mutations in target protein PiORP1 of fluoxapiprolin in *Phytophthora infestans* . J Agric Food Chem 70:4881–4888 (2022). 35416662 10.1021/acs.jafc.1c08199

[108] Stenzel K and Vors JP , Sterol biosynthesis inhibitors, in Modern Crop Protection Compounds, Vol. 2, Fungicides, 3rd edn, ed. by Jeschke P , Witschel M , Krämer W and Schirmer U . VCH‐Wiley, Weinheim, Germany, pp. 797–844 (2019).

[109] Tesh SA , Tesh JM , Fegert I , Buesen R , Schneider S , Mentzel T *et al*., Innovative selection approach for a new antifungal agent mefentrifluconazol (Revisol) and the impact on ist toxicity profile. Regulat Toxicol Pharmacol 106:152–168 (2017). 10.1016/j.yrtph.2019.04.009 31026541

[110] Gebhardt J , Ehresmann M , Chiodo T , Viertelhaus M and Goetz R , Process for the Preparation of Substituted Oxiranes and Triazoles. PCT Int. Appl. WO 2016/005211, BASF S.E. (2016).

[111] Xu S , Shen J , Lang H , Zhang L , Fang H and Yu Y , Triazole resistance in *Aspergillus fumigatus* exposed to new chiral fungicide mefentrifluconazole. Pest Manag Sci 79:560–568 (2023). 36205310 10.1002/ps.7224

[112] Tong Z , Meng DD , Zhang WY , Jin L , Yi XT , Dong X *et al*., Mechanism insights into the enantioselective bioactivity and fumonisin biosynthesis of mefentrifluconazole to *Fusarium verticillioides* . J Agric Food Chem 72:9044–9053 (2024). 10.1021/acs.jafc.4c01336 38607803

[113] Liu T , Ren X , Fang J , Yu Z and Wang X , Multiomics sequencing and AlphaFold2 analysis of the stereoselective behavior of mefentrifluconazole for bioactivity improvement and risk reduction. Environ Sci Technol 57:21348–21357 (2023). 38051155 10.1021/acs.est.3c05327

[114] Babij NR , McCusker EO , Whiteker GT , Sane N , Tu S , Li X *et al*., Process for synthesis of picolinamides. PCT int. appl. WO 2021/076681 Corteva Agriscience LLC (2021).

[115] Zakharycheva VV and Martsynkevich AM , Development of novel pyridine‐based agrochemicals: a review. Adv Agrochem (2024). 10.1016/j.aac.2024.10.002.

[116] Hoekstra WJ , Wirth DD , Ehiwe T and Bonnaud T , Antifungal Compounds and Processes for Making. PCT Int. Appl. WO 2016/187201. Johnson Matthey PLC Mycovia Pharmaceuticals Inc., (2016).

[117] Kandasamy R , London D , Stam L , von Deyn W , Zhao X , Salgado VL *et al*., Afidopyropen: new and potent modulator of insect transient receptor potential channels. Ins Biochem Molecul Biol 84:32–39 (2017). 10.1016/j.ibmb.2017.03.005 28347703

[118] Kandasamy R , Costea PI , Stam L and Nesterov A , TRPV channel nanchung and TRPA channel water witch form insecticide‐activated complexes. Ins Biochem Molecul Biol 149:103835 (2022). 10.1016/j.ibmb.2022.103835 36087889

[119] Koradin C , Schröder H , Oyama K and Ōmura S , Chemistry and biology connected: the development of Inscalis, in Recent Highlights in the Discovery and Optimization of Crop Protection Products, ed. by Maienfisch P and Mangelinckx S . Amsterdam, Academic Press, pp. 231–239 (2021).

[120] Goto K , Horikoshi R , Mitomi M , Oyama K , Hirose T , Sunazuka T *et al*., Synthesis and insecticidal efficacy of pyripyropene derivatives. Part II – invention of afidopyropen. J Antibiot 72:661–681 (2019). 10.1038/s41429-019-0193-9 31222131

[121] Shafi MS , Iqbal N , Naqqash MN , Saeed S , Usman M , Abid AD *et al*., Transgenerational effect of afidopyropen on *Bemisia tabaci* Gennadius (Homoptera: Aleyrodidae). Sci Rep 13:19988 (2023). 37968272 10.1038/s41598-023-46479-0 PMC10651898

[122] Hodges D , Discovery, research and development of Axalion® active insecticide: dimpropyridaz. Pest Manag Sci (2024). 10.1002/ps.8385. PMC11981975 39320022

[123] Shang J , Dong W , Fang H , Wang C , Yang H , Chen Z *et al*., Effects of dimpropyridaz on feeding behavior, locomotivity and biological parameters of *Aphis gossypii* . Pestic Biochem Physiol 197:105694 (2023). 38072549 10.1016/j.pestbp.2023.105694

[124] Spalthoff C , Salgado VL , David MD , Hehlert P , Balu N , Huang H *et al*., The novel pyridazine pyrazolecarboxamide insecticide dimpropyridaz inhibits chordotonal organ function upstream of TRPV channels. Pest Manag Sci 79:1635–1649 (2023). 36622360 10.1002/ps.7352

[125] Technical Guide , Efficon® insecticide − powered by Axalion® active, a new standard for piercing and sucking pest control. BASF 1–24 (2023).

[126] Salgado VL , Schnatterer S and Holmes KA , GABA‐gated chloride channel antagonists (Fiproles), in Modern Crop Protection Compounds, Vol. 3, Insecticides, 3rd edn, ed. by Jeschke P , Witschel M , Krämer W and Schirmer U . VCH‐Wiley, Weinheim, Germany, pp. 1449–1478 (2019).

[127] Asahi M , Kobayashi M , Kagami T , Nakahira K , Furukawa Y and Ozoe Y , Fluxametamide: a novel isoxazoline insecticide that acts via distinctive antagonism of insect ligand‐gated chloride channels. Pestic Biochem Physiol 151:67–72 (2017). 10.1016/j.pestbp.2018.02.002 30704715

[128] Mita T , Furukawa Y , Iwasa M , Kikuchi T and Komoda K , Studies on a novel insecticide, fluxametamide, in Recent Highlights in the Discovery and Optimization of Crop Protection Products, ed. by Maienfisch P and Mangelinckx S . Amsterdam, Academic Press, pp. 157–163 (2021).

[129] Cassayre J , Smejkal T , Blythe J , Hoegger P , Renold P , Pitterna T *et al*., The discovery of isocycloseram: a novel isoxazoline insecticide, in Recent Highlights in the Discovery and Optimization of Crop Protection Products, ed. by Maienfisch P and Mangelinckx S . Amsterdam, Academic Press, pp. 165–212 (2021).

[130] Blythe J , Earley FGP , Hirst EA , Piekarska‐Hack K , Goodchild JA , Elke Hillesheim E *et al*., The mode of action of isocycloseram: a novel isoxazoline insecticide. Pestic Biochem Physiol 187:105217 (2022). 36127059 10.1016/j.pestbp.2022.105217

[131] Zanetti R , Sanches JJ , Wenzel AVA , Haddi K , Ferreira H and Santos LV , Isocycloseram: a new active ingredient for leaf‐cutting ants control. PLoS One 19:e0300187 (2024). 38722866 10.1371/journal.pone.0300187 PMC11081378

[132] Cordova D , Zhang W , Holyoke CW Jr , Barry JD , SinghV AIB *et al*., Triflumezopyrim: A mesoionic insecticide, in Modern Crop Protection Compounds, Vol. 3, Insecticides, 3rd edn, ed. by Jeschke P , Witschel M , Krämer W and Schirmer U . VCH‐Wiley, Weinheim, Germany, pp. 1384–1400 (2019).

[133] Huang H , Dickhaut J , Weisel M , Mao L , Rankl N , Takeda H *et al*., Discovery and biological characterization of a novel mesoionic insecticide fenmezoditiaz. Pest Manag Sci (2024). 10.1002/ps.8108. PMC11981979 38554053

[134] Hirata K , Kudo K , Amano T and Kawaguchi M , Effects of the novel acaricide acynonapyr on the calcium‐activated potassium channel. Pestic Biochem Physiol 204:106074 (2024). 39277387 10.1016/j.pestbp.2024.106074

[135] Hamamoto I , Kawaguchi M , Nakamura T , Yano M , Koizumi K and Takahashi J , Discovery of a novel acaricide, acynonapyr. J Pestic Sci 48:202–210 (2023). 38090213 10.1584/jpestics.D23-028 PMC10710939

[136] Desaeger J , Wram C and Zasada I , New reduced‐risk agricultural nematicides – rationale and review. J Nematol 52:e2020–e2911 (2020). 10.21307/jofnem-2020-091 PMC8015323 33829179

[137] Burns AR , Baker RJ , Kitner M , Knox J , Cooke B , Volpatti JR *et al*., Selective control of parasitic nematodes using bioactivated nematicides. Nature 618:102–109 (2023). 37225985 10.1038/s41586-023-06105-5

[138] O'Sullivan AC , Mondiere RJG , Loiseleur O , Smejkal T , Luksch T , Jeanguenat A *et al*., 4‐membered ring carboxamides used as nematicides. PCT int. appl. WO 2015/003951. Syngenta AG (2015).

[139] Heydari F , Rodriguez‐Crespo D and Wicky C , The new nematicide cyclobutrifluram targets the mitochondrial succinate dehydrogenase complex in Caenorhabditis elegans. J Dev Biol 11:39 (2023). 10.3390/jdb11040039. 37873747 PMC10594496

