# Supplementary material for: The continuing significance of chiral agrochemicals
Source: Pest Manag Sci. 2025 Jan 17;81(4):1697–716. doi: 10.1002/ps.8655 (PMC11906909; doi:10.1002/ps.8655)
Supplement: Supplementary file 1 — Figure S1. Chiral development candidate herbicides: protoporphyrinogen IX oxidase (PPO) inhibitor flufenoximacil, auxin inhibitor fluchchloraminopyr and its proherbicide fluchchloraminopyr‐tefuryl, 4‐hydroxy‐phenylpyrovate dioxygenase (4‐HPPD) inhibitor flusulfinam as (R)‐enantiomer (major component), fatty acid thioesterase (FAT) inhibitor cinflubrolin as mixture of the (1S,2R,4R)‐ and (1R,2S,4S)‐stereoisomers, and the plant tubulin polymerization inhibitor icafolin and its proherbicide icafolin‐methyl as mixture of the (2R,4R)‐ and (2S,4S)‐stereoisomers. (ISO‐proposed common names.) Figure S2. Chiral development candidate fungicides: fungicidal quinone inside inhibitors (QiIs) metarylpicoxamid as (1S,2S,4S)‐stereoisomer, and the racemic sterol biosynthesis inhibitor (SBI) fluoxytioconazole. (ISO‐proposed common names.) Figure S3. Chiral development candidate insecticdes: insecticidal mesoinonic triflumezopyrim as nAChR competitive modulator from the IRAC MoA sub‐group 4E has been the starting point for development of fenmezoditiaz as (R)‐enantiomer. (ISO‐proposed common name.) [file PS-81-1697-s001.docx]

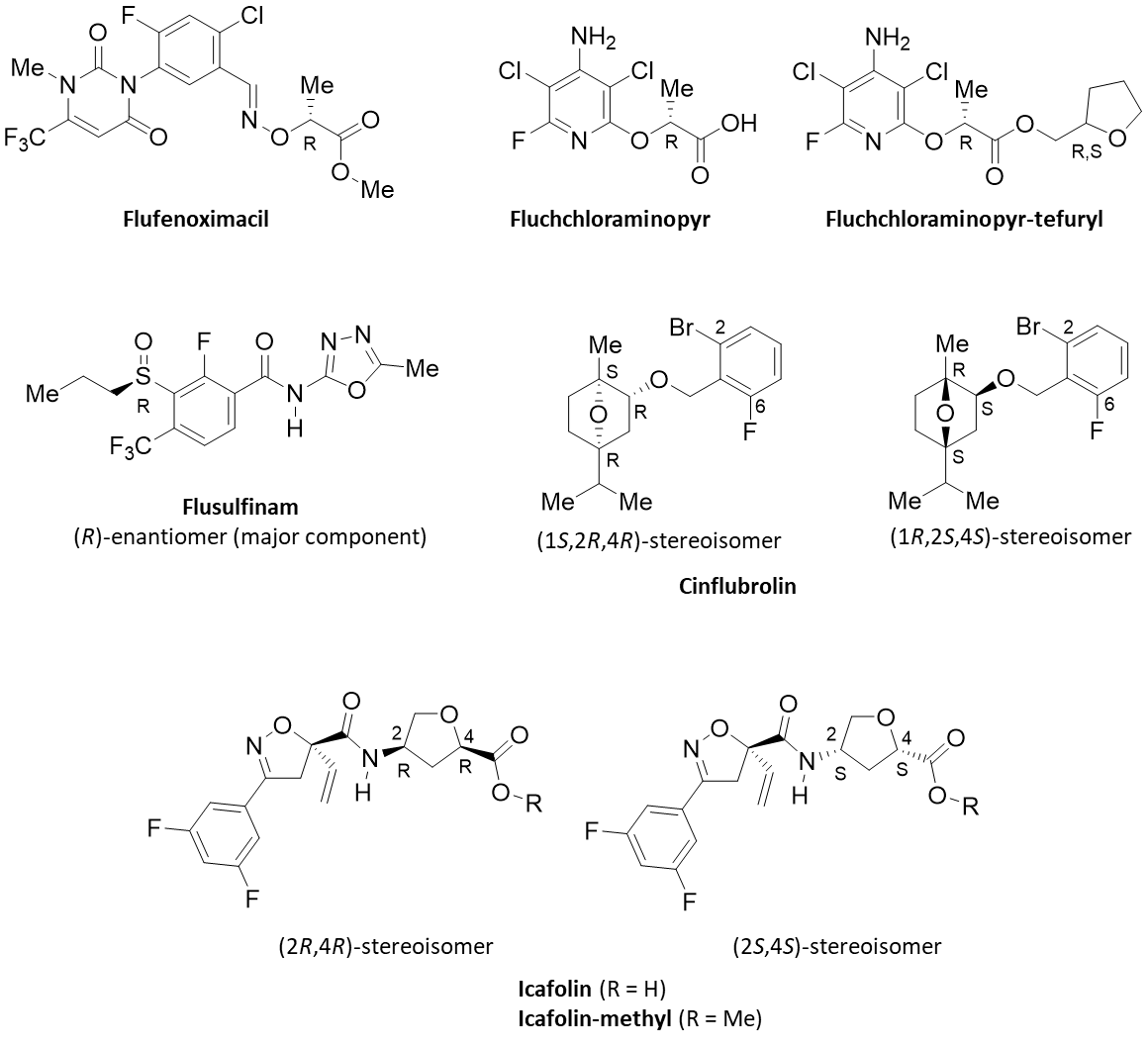


**Figure S1**. Chiral development candidate herbicides: protoporphyrinogen IX oxidase (PPO) inhibitor flufenoximacil, auxin inhibitor fluchchloraminopyr and its proherbicide fluchchloraminopyr-tefuryl, 4-hydroxy-phenylpyrovate dioxygenase (4-HPPD) inhibitor flusulfinam as (*R*)-enantiomer (major component), acyl-acyl thioesterase (FAT) inhibitor cinflubrolin as as mixture of the (1*S*,2*R*,4*R*)- and (1*R*,2*S*,4*S*)-stereoisomers, and the plant tubulin polymerization inhibitor icafolin and its proherbicide icafolin-methyl as mixture of the (2*R*,4*R*)- and (2*S*,4*S*)-stereoisomers. (ISO-proposed common names)


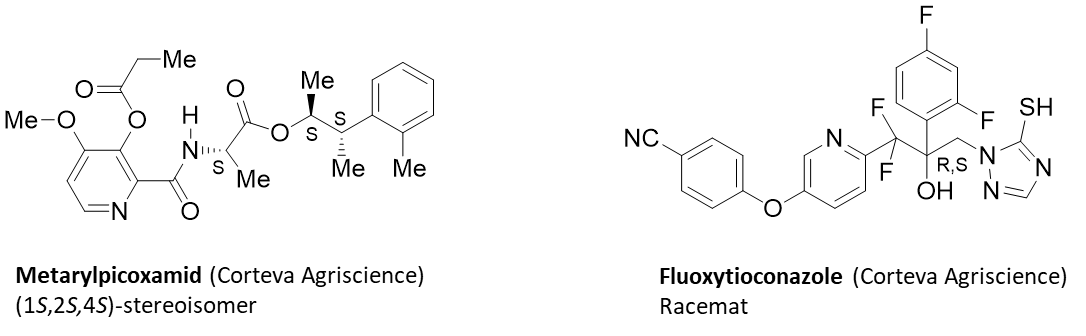


**Figure S2**. Chiral development candidate fungicides: fungicidal quinone inside inhibitors (QiIs) metarylpicoxamid as (1*S*,2*S*,4*S*)-stereoisomer, and the racemic sterol biosythesis inhibitor (SBI) fluoxytioconazole. (ISO-proposed common names)


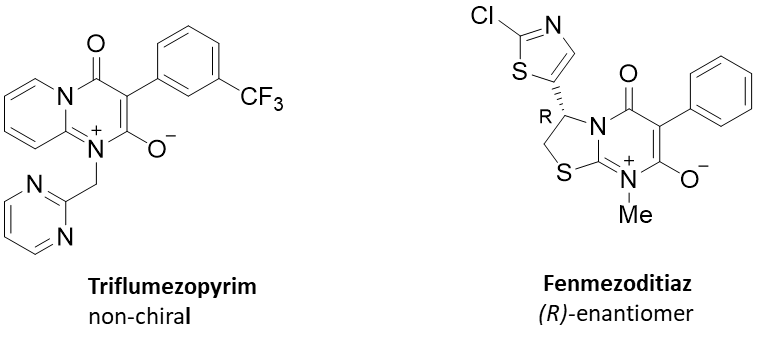


**Figure S3.** Chiral development candidate insecticdes: insecticidal mesoinonic triflumezopyrim as *n*AChR competitive modulator from the IRAC MoA sub-group 4E has been the starting point for development of fenmezoditiaz as *(R)-*enantiomer (ISO-proposed common name).
