# Supplementary material for: The continuing significance of chiral agrochemicals
Source: Pest Manag Sci. 2025 Jan 17;81(4):1697–716. doi: 10.1002/ps.8655 (PMC11906909; doi:10.1002/ps.8655)
Supplement: Supplementary file 2 — Table S1. Chiral separation, preparation and detection methods of enantiomers. Table S2. Catalytic asymmetric syntheses of agrochemical intermediates and final products launched between 2018 and 2023. Table S3. The provision of starting materials for chiral fungicides and insecticides is based on fermentation of natural products. [file PS-81-1697-s002.zip › ps8655-sup-0002-TablesS1,S3.docx]

**Table S1.** Chiral separation, preparation and detection methods of enantiomers.

| **Common name** | **Use** | **Stereoisomers** | **Methode** | **Section** | **Reference** |
| --- | --- | --- | --- | --- | --- |
| Fluindapyr | Fungicide | (R) and (S)-enantiomers | LC ^a^ | 5.1.2 | 27 |
| Mefentrifluconazole | Fungicide | *(R)-(-)-* and  *(S)-(+)-*enantiomers | UPLC-MS/MS | 5.4.1 | 28 |
| Fluxametamide | Insecticide | *(R)-(-)-* and  *(S)-(+)-*enantiomers | SFC-MS/MS | 6.3.1 | 29 |

^a^ Daicel Chiralpak AD-3R, OX-3R and IK-3.

**Table S3.** The provision of starting materials for chiral fungicides and insecticides is based

on fermentation of natural products.

| **Common name** | **Use** | **Strain** | **Natural Product** | **Section** | **Reference** |
| --- | --- | --- | --- | --- | --- |
| Fenpicoxamid | Fungicide | *Streptomyces* sp. 517-02 | UK-2A | 5.2.1 | 43 |
| Afidopyropen | Insecticide | *Aspergillus fumigatus*  FO-1289  *Penicillium griseofulvum* F1959  *Penicillium coprobium* PF1169 | Pyripyropen A | 6.1.1 | 44  45  46 |
