# Supplementary material for: The continuing significance of chiral agrochemicals
Source: Pest Manag Sci. 2025 Jan 17;81(4):1697–716. doi: 10.1002/ps.8655 (PMC11906909; doi:10.1002/ps.8655)
Supplement: Supplementary file 2 — Table S1. Chiral separation, preparation and detection methods of enantiomers. Table S2. Catalytic asymmetric syntheses of agrochemical intermediates and final products launched between 2018 and 2023. Table S3. The provision of starting materials for chiral fungicides and insecticides is based on fermentation of natural products. [file PS-81-1697-s002.zip › ps8655-sup-0003-TableS2.docx]

**Table S2.** Catalytic asymmetric syntheses of agrochemical intermediates and final products launched between 2018 and 2023.

| **Common Name** | **Use** | **First stereoselective reaction step** | **Catalyst** | **Section** | **Reference** |
| --- | --- | --- | --- | --- | --- |
| Cinmethylin | Herbicde | 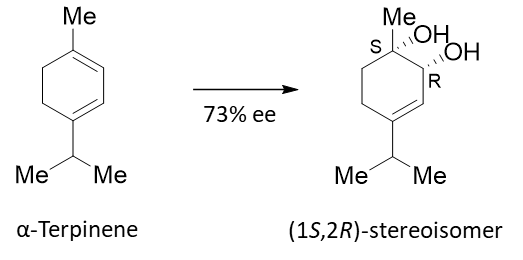 | AD-mix-β | 4.2.1 | 34 |
|  |  |  |  |  |  |
| Tetflupyrolimet | Herbicide | 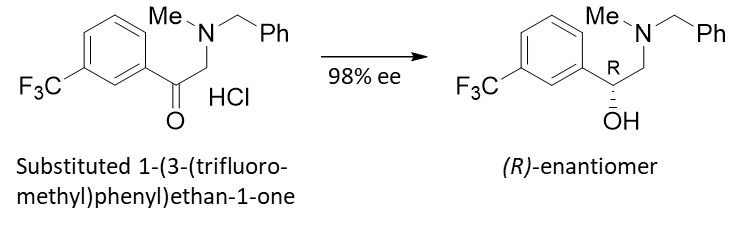 | Ru-cat. | 4.3.1 | 35, 36 |
| Cyclobutrifluram | Fungicide  Nematicide | 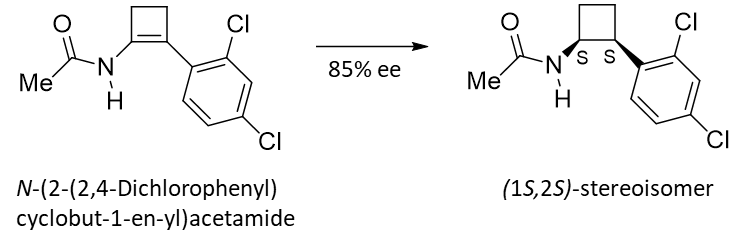 | [Rh(cod)_2_]OTf  Josiphos SL-J505-1 ^a^ | 5.1.3  8.1 | 36, 37 |
| Isocycloseram | Insecticide | 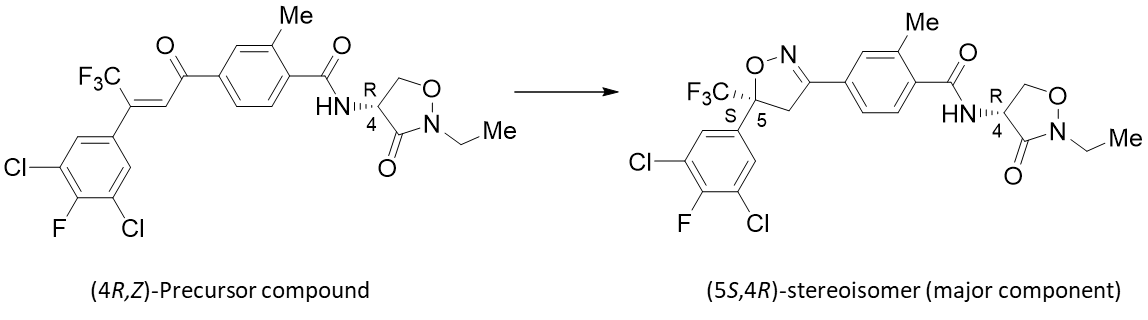 | Anthracene-bridged dimer ^b^ | 6.3.2 | 38, 39 |

^a^ (*R*)-1-[(SP)-2-(Di-tert-butylphosphino)ferrocenyl]ethylbis(2-methylphenyl)phosphine.

^b^ (8α,9R)-(8’’α,9’’R)-1,1’’-[9,10-anthracenediylbis(methylene)]bis[9-hydroxy-6′-methoxy-cinchonanium dibromide.
